# Growth inhibition of cytosolic Salmonella by caspase-1 and caspase-11 precedes host cell death

**DOI:** 10.1038/ncomms13292

**Published:** 2016-11-03

**Authors:** Teresa L. M. Thurston, Sophie A. Matthews, Elliott Jennings, Eric Alix, Feng Shao, Avinash R. Shenoy, Mark A. Birrell, David W. Holden

**Affiliations:** 1 MRC Centre for Molecular Bacteriology and Infection, Imperial College London, Flowers Building Exhibition Road, London SW7 2AZ, UK; 2 National Institute of Biological Sciences, 7 Science Park Road, Zhongguancun Life Science Park, Beijing 102206, PR China; 3 Respiratory Pharmacology, Pharmacology & Toxicology Section, Imperial College London, Sir Alexander Fleming Building, London SW7 2AZ, UK

## Abstract

Sensing bacterial products in the cytosol of mammalian cells by NOD-like receptors leads to the activation of caspase-1 inflammasomes, and the production of the pro-inflammatory cytokines interleukin (IL)-18 and IL-1β. In addition, mouse caspase-11 (represented in humans by its orthologs, caspase-4 and caspase-5) detects cytosolic bacterial LPS directly. Activation of caspase-1 and caspase-11 initiates pyroptotic host cell death that releases potentially harmful bacteria from the nutrient-rich host cell cytosol into the extracellular environment. Here we use single cell analysis and time-lapse microscopy to identify a subpopulation of host cells, in which growth of cytosolic *Salmonella* Typhimurium is inhibited independently or prior to the onset of cell death. The enzymatic activities of caspase-1 and caspase-11 are required for growth inhibition in different cell types. Our results reveal that these proteases have important functions beyond the direct induction of pyroptosis and proinflammatory cytokine secretion in the control of growth and elimination of cytosolic bacteria.

## Abstract (web summary)

Inflammatory caspases restrict microbial growth by inducing cytokine production and pyroptosis, but other caspase-induced mechanisms are thought to contribute. Here the authors use time-lapse microscopy of single cells to show that caspase1/11 has anti-Salmonella functions that occur in advance of cell death induction.

Effective mammalian immune responses to bacterial pathogens depend on the detection of bacterial-derived molecules in both extracellular and intracellular environments by pattern recognition receptors (PRRs). Toll-like receptor (TLR) family members detect bacterial molecules in the extracellular environment, initiating activation of multiple transcription factors including nuclear factor κB, interferon regulatory factor and activator protein 1 (AP-1) family members^1^. The resulting changes in gene expression drive immune responses, including the production of interferons, microbicidal proteins and pro-inflammatory cytokines such as pro-interleukin-1β (IL-1β) (refs 2, 3). Proteins of the nucleotide-binding oligomerization domain (NOD)-like receptor (NLR; nucleotide-binding domain leucine-rich repeat containing receptor) family detect intracellular bacterial products that are either shed or delivered by secretion systems into the host cell cytosol, as well as other stress or danger-associated signals. On sensing bacterial infection, some NLRs and AIM2-like receptors (ALRs), activate caspase-1 by forming multi-protein complexes called inflammasomes^4^,^5^. Caspase-1 is the prototype of a family of inflammatory caspases that also includes caspase-11 (caspase-4/−5 in humans) and caspase-12 (ref. 6).

*Salmonella enterica* serovar Typhimurium (Salmonella) has been used extensively, as a model Gram-negative pathogen to help elucidate the molecular mechanisms of virulence and immunity. It replicates within a variety of host cells in membrane-bound compartments termed Salmonella-containing vacuoles (SCVs). However, it can also enter the host cell cytosol in different ways. First, the SPI-1 encoded type III secretion system (T3SS) that enables host cell invasion also destabilizes the SCV membrane of approximately 10% of bacteria shortly after host cell entry, leading to a subpopulation of cytosolic Salmonella^7^,^8^. Second, even if wild-type (WT) bacteria are grown to stationary phase (where the SPI-1 T3SS is down regulated) and enter macrophages through phagocytosis, ∼5% of resulting vacuoles undergo rupture^9^. The proportion of cytosolic Salmonella within macrophages can be enhanced using mutant strains. Following the acidification of the SCV lumen, Salmonella expresses its SPI-2-encoded T3SS that delivers effector proteins across the SCV membrane into the host cell. Some of these effectors, including SifA, act collectively to ensure vacuolar membrane stability^10^. The vacuolar membrane of a *sifA* mutant is unstable and >50% of bacteria are released into the macrophage cell cytosol from ∼6 h following uptake^10^.

*Casp1/11*^*−/−*^ mice display increased susceptibility to many bacterial pathogens, including Salmonella^11^,^12^. These mice were originally described as caspase-1 knockouts but were subsequently found to also contain a germline mutation of caspase-11 (ref. 13). Since then, several studies have helped to delineate the functions of caspase-1 and caspase-11.

Caspase-1, constitutively present in macrophages, requires stimulation of the NLRC4 (NLR family CARD-domain containing protein 4) and NLRP3 (NOD, LRR and pyrin domain-containing 3) receptors for activation during Salmonella infection. Caspase-11 on the other hand is transcriptionally upregulated through TLR4-TRIF and STAT signalling^14^,^15^,^16^. Both caspase-1 and caspase-11 mediate pro-inflammatory immune responses and also initiate a form of inflammatory cell death termed pyroptosis^17^ in response to Salmonella^16^. On detection of cytosolic lipopolysaccharide (LPS)^13^,^18^,^19^ caspase-11 activates caspase-1 through a non-canonical pathway involving the downstream activities of NLRP3 and the adaptor protein ASC (apoptosis-associated speck-like protein)^13^, resulting in processed IL-1β and IL-18. In contrast, cytosolic LPS-induced pyroptosis and IL-1α release requires caspase-11 but not caspase-1 activity^13^,^20^.

In mouse models of infection, caspase-1 but not caspase-11 contributes to the control of WT Salmonella growth in systemic organs such as the liver and spleen^16^. Macrophage pyroptosis releases Salmonella into the extracellular matrix. Subsequent phagocytosis by recruited neutrophils is thought to eliminate bacteria via reactive oxygen species^16^,^21^,^22^,^23^. Pyroptosis and subsequent recruitment of neutrophils have also been proposed to explain the protective effect of caspase-11 against the Salmonella *sifA* mutant^23^. Finally, cell death of infected epithelial cells is followed by their extrusion and clearance from the gut lumen^24^,^25^, thereby controlling the bacterial burden.

Thus, host cell death appears to be an important means of expelling Salmonella from the nutrient rich host cell cytosol. However, in several studies the overall level of cytotoxicity of host cells infected with the *sifA* mutant did not exceed 30% (refs 18, 23, 26). In addition, the *sifA* mutant was reported to be defective for replication in the cytosol of macrophages and 3T3 fibroblasts^27^,^28^. Caspase-1 and 11 have also been implicated in non-pyroptotic growth control of vacuolar bacterial pathogens, revealing the existence of additional functions for these proteases^29^,^30^,^31^,^32^. Altogether, these findings suggest that non-pyroptotic mechanism(s) might also contribute to the control of bacterial growth in the host cell cytosol.

Using single cell analysis and time-lapse microscopy, we identify a subpopulation of host cells in which growth of cytosolic Salmonella is inhibited independently or before the onset of cell death. Since this requires the activity of caspase-1 and caspase-11, our results reveal additional functions for these proteases in the control of cytosolic bacteria.

## Results

### Caspase-mediated growth inhibition of cytosolic Salmonella

**Figure 1:**
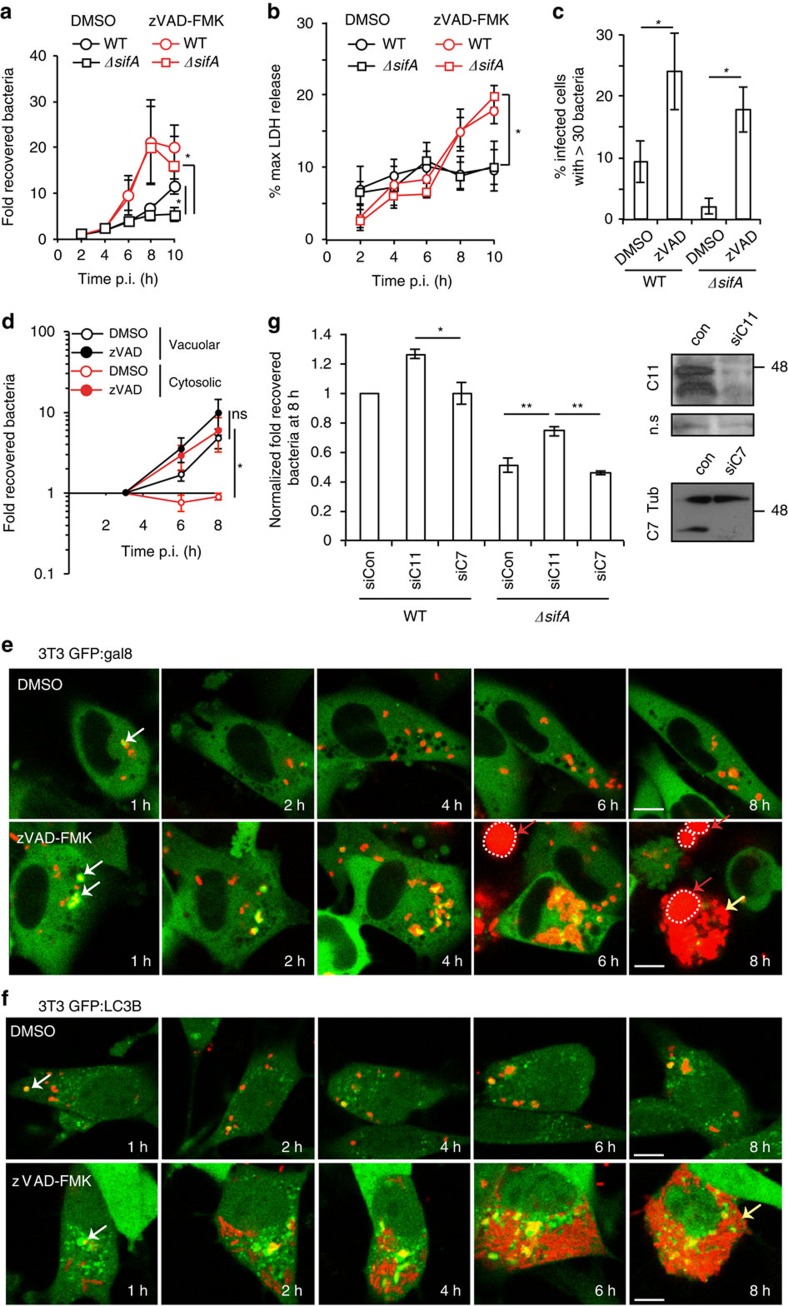
Caspase-dependent inhibition of cytosolic Salmonella in 3T3 fibroblasts. (**a**) Bacterial replication in 3T3 fibroblasts pre-treated with DMSO or zVAD-FMK and infected with WT or *ΔsifA* Salmonella was determined by enumeration of colony-forming units (CFUs) after cell lysis at the indicated times post-invasion (p.i.). (**b**,**c**) Cells treated as in (**a**) were analysed for LDH release over time (**b**) or quantified for the number of cells with >30 bacteria at 8 h (**c**). (**d**) Following pre-treatment with DMSO or zVAD-FMK, WT Salmonella-infected 3T3 fibroblasts were vehicle control treated or exposed to chloroquine (CQ) from 1.5 to 3 h. The numbers of surviving bacteria for the cytosolic (CQ resistant) population and vacuolar (total—CQ resistant) populations were determined by CFU at the indicated times p.i. (**e**,**f**) DMSO or zVAD-FMK pre-treated 3T3 fibroblasts expressing GFP-galectin-8 (**e**) or GFP-LC3B (**f**) were infected with mCherry-expressing WT Salmonella and imaged over 8 h in the presence of PI. White arrows—bacteria associated with ruptured vacuoles. Red arrows—PI-positive nuclei (red surrounded by white dotted line). Yellow arrows—bacteria that have undergone replication. (**g**) siRNA-treated 3T3 fibroblasts were infected with WT or *ΔsifA* Salmonella. Fold recovered bacteria determined by CFUs was calculated from 2 to 8 h and normalized to the WT-infected siCon condition. After siRNA treatment, protein extracts were analysed by immunoblotting for caspase-7 (C7), caspase-11 (C11) and Tubulin (Tub), n.s denotes a non-specific band, serving as loading control. Data represent mean and s.e.m. of three (**a**–**d**) or four (**g**) independent experiments. Student's *t*-test, *P<0.05, ^**^P<0.01. Scale bar, 10 μM.

3T3 fibroblasts are relatively non-permissive for the growth of *sifA* mutant Salmonella (*ΔsifA)*^27^ (Fig. 1a), but the mechanism for this is unclear. In DMSO treated samples, the similar levels of lactate dehydrogenase (LDH) released from fibroblasts invaded by WT or *ΔsifA* Salmonella (Fig. 1b) suggest that host cell death is not sufficient to explain the reduced growth of *ΔsifA* bacteria (Fig. 1a). To explore the contribution of caspases in the growth inhibition of cytosolic Salmonella we exposed 3T3 fibroblasts to the irreversible pan-caspase inhibitor zVAD-FMK. Addition of zVAD-FMK resulted in greater LDH release from cells infected with either WT or *ΔsifA* Salmonella from 6 h post-invasion (p.i.) (Fig. 1b). Nevertheless, intracellular bacterial growth of *ΔsifA* Salmonella, measured by CFU (10 h p.i.), increased significantly in cells treated with zVAD-FMK (Fig. 1a). Microscopic analysis revealed that inhibition of caspases led to an increase in the % of infected cells harbouring >30 bacteria, for both WT and *ΔsifA* Salmonella (Fig. 1c).

Following SPI-1 T3SS-mediated invasion of cells, between 10 and 20% of vacuoles were ruptured (Supplementary Fig. 1B), yielding a naturally occurring population of cytosolic WT bacteria^7^. *ΔsifA* bacteria yield a greater population of cytosolic bacteria, as a result of SPI-1 T3SS-mediated instability of the early vacuole (1–2 h) and deregulated activities of SPI-2 T3SS effectors (4–6 h onwards)^27^. To determine if caspase-mediated inhibition of WT Salmonella affected cytosolic and/or vacuolar bacteria, bacterial growth was analysed after exposure of infected cells to chloroquine (CQ), which accumulates to bactericidal concentrations selectively within acidic endosomes and vacuoles, including the SCV (refs 33, 34). A *prgH* mutant is defective for SPI-1 function but can enter non-phagocytic cells if it carries a plasmid expressing *Yersinia* Invasin^35^. As non-SPI-1-mediated entry results in decreased vacuole rupture in epithelial cells^7^, the Invasin-producing *prgH* mutant was used as a control to establish a concentration of CQ sufficient to kill >99% of bacteria. At this concentration, ∼5% (±2%) of WT Salmonella survived, and only underwent replication when zVAD-FMK was added prior to infection (Fig. 1d, cytosolic population). In contrast, when only the vacuolar population (total CFU counts—cytosolic population) was analysed (Fig. 1d, vacuolar population), there was no statistical difference in CFU following the addition of zVAD-FMK. Furthermore, zVAD-FMK had no effect on the recovery of the Invasin-producing *prgH* mutant, confirming that caspase inhibition does not influence vacuolar Salmonella (Supplementary Fig. 1A). Therefore, when only cytosolic bacteria were analysed, a strong caspase-dependent inhibition on bacterial numbers was detected.

To visualize cytosolic replication of WT bacteria after inhibition of caspase activity, infected 3T3 cells expressing GFP-tagged galectin-8 (a marker of vacuole integrity^36^) were imaged over time in the presence of the membrane impermeant dye propidium iodide, so that viable cells could be distinguished from dying cells. zVAD-FMK did not affect recruitment of galectin-8 or LC3B (an autophagy protein that is frequently recruited to bacteria following vacuole rupture^7^) to ruptured SCVs (Supplementary Fig. 1B,C). Pan-caspase inhibition resulted in a dramatic increase in Salmonella replication in cells containing ruptured SCVs (Fig. 1e). Importantly, when bacterial replication was observed, it preceded the uptake of PI (Fig. 1e). Time-lapse imaging in GFP-LC3B-expressing fibroblasts following exposure to zVAD-FMK confirmed the striking degree of bacterial replication, even in cells where LC3B was recruited to bacteria (Fig. 1f). As zVAD-FMK does not influence SCV stability, our results strongly suggest that caspases inhibit growth of WT and *ΔsifA* Salmonella in the cytosol of 3T3 fibroblasts.

To investigate the contribution of individual caspases, we tested more specific peptide-based inhibitors of caspases and siRNA-mediated depletion of caspase-11 or caspase-7. None of these peptide inhibitors had an effect on bacterial growth (Supplementary Fig. 1D). Only knockdown of caspase-11 resulted in increased WT and *ΔsifA* bacterial numbers (Fig. 1g).

### Caspase-11 production in MEFs reduces bacterial replication

**Figure 2:**
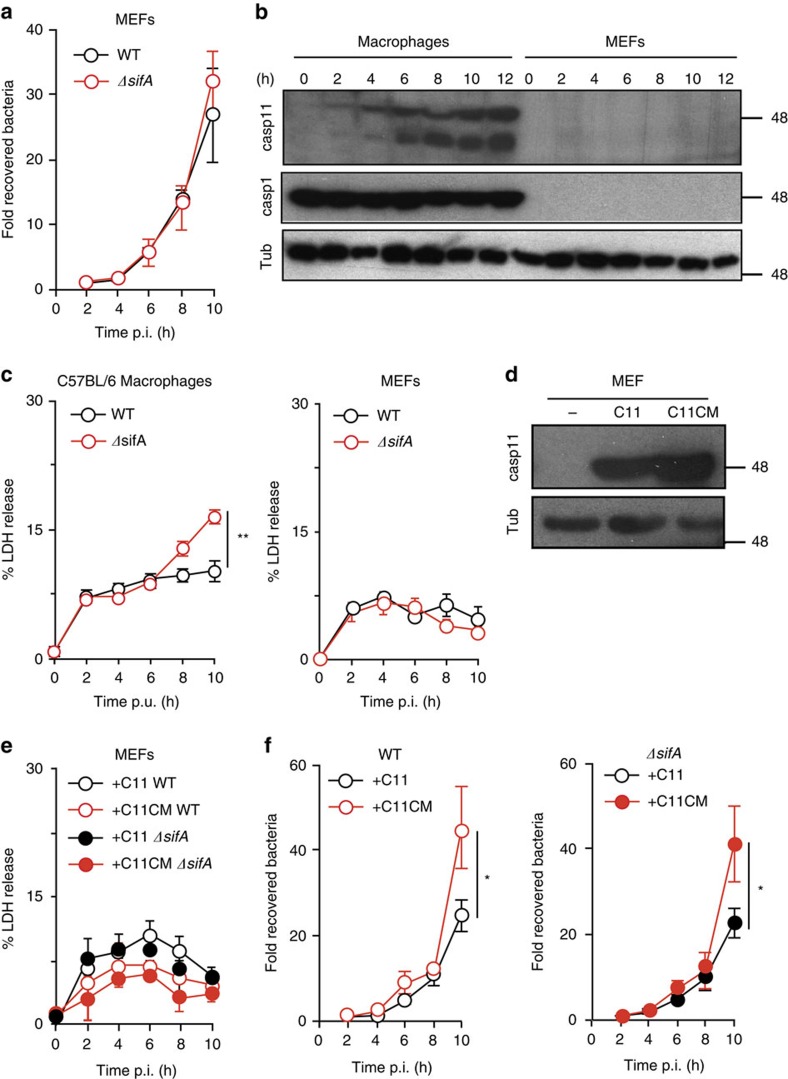
Expression of caspase-11 in MEFs reduces growth of Salmonella. (**a**) MEFs were infected with WT or *ΔsifA* Salmonella then lysed at the indicated time points. Bacterial CFUs were counted on LB agar and normalized to CFUs at 2 h p.i. (**b**) Protein extracts from iBMDMs or MEFs, infected with *ΔsifA* Salmonella for the times indicated, were immunoblotted for caspase-11 (Casp11), caspase-1 (Casp1) or Tubulin (Tub) as a control. (**c**) Cytotoxicity of iBMDMs or MEFs, infected with WT or *ΔsifA* Salmonella, was quantified by release of LDH at the indicated times. post-uptake (p.u.). (**d**) MEFs, retrovirally transduced with vector encoding caspase-11 (C11) or a catalytic mutant (C11CM) were immunoblotted with caspase-11 or Tubulin antibodies and compared with control-treated cells. (**e**,**f**) Cells described in (**d**) were infected with WT or *ΔsifA* Salmonella prior to kinetic analysis of LDH release (**e**) and enumeration of bacterial CFUs on LB agar (**f**). Data are the mean and s.e.m. from three experiments (**a**,**c**,**e**,**f**) or are representative of two experiments (**b**,**d**). Student's *t*-test, *P<0.05.

The Salmonella *ΔsifA* mutant replicates in the cytosol of HeLa cells^27^,^37^ and certain mouse embryonic fibroblast lines (MEFs) also appear permissive for the growth of this strain (Fig. 2a;^38^). Interestingly, in MEFs this was correlated with the absence of detectable caspase-11 (Supplementary Fig. 2A) and almost 100-fold less basal caspase-11 messengerRNA (mRNA) (but not caspase-7), compared with RAW macrophages, immortalized bone-marrow-derived macrophages (iBMDMs) or 3T3 fibroblasts (Supplementary Fig. 2A,B). Transcriptional upregulation of caspase-11 occurs following activation of TLR4 by extracellular LPS^14^,^15^. MEFs infected with Salmonella failed to induce detectable levels of caspase-11 (Fig. 2b), which might explain the lack of *ΔsifA* Salmonella infection-induced cell death in MEFs compared with iBMDMs (Fig. 2c).

To test if the permissiveness of MEFs was due to the low levels of caspase-11, these cells were transduced to express either WT caspase-11 (C11) or catalytic mutant caspase-11 where the critical cysteine^39^ was replaced for glycine at position 254 (C11CM) (Fig. 2d). Neither form of caspase-11 affected LDH release following invasion by WT or *ΔsifA* Salmonella (compare Fig. 2c right panel with Fig. 2e). However, catalytically active caspase-11 partially reduced the replication of both WT and *ΔsifA* Salmonella (Fig. 2f). Therefore, when introduced into MEFs, caspase-11 restricts intracellular bacterial replication in the absence of detectable host cell death.

### Caspase-11 restrains *ΔsifA* mutant growth in macrophages

**Figure 3:**
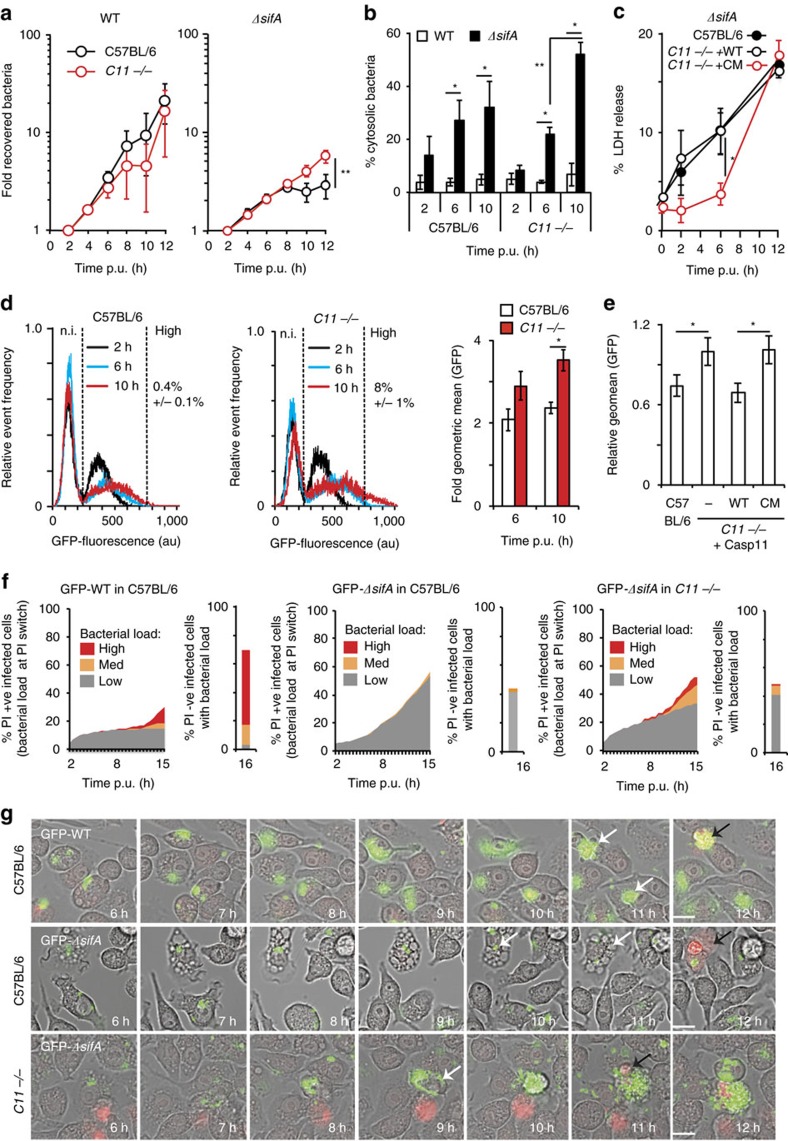
*ΔsifA* growth within macrophages is partially inhibited by caspase-11. (**a**) Kinetics of intracellular growth of wild-type (WT) or *ΔsifA* Salmonella after infection of the indicated iBMDMs. Following host-cell lysis at the indicated time-points, intracellular bacteria were enumerated by CFU on LB agar and normalized to CFU at 2 h. (**b**) Quantification of cytosolic bacteria in iBMDMs by immunofluorescence microscopy after selective permeabilisation of the plasma membrane at the indicated times. (**c**) LDH release from C57BL/6 or *Casp11*^*−/−*^ iBMDMs complemented with WT or catalytic mutant caspase-11 (CM) infected with *ΔsifA* Salmonella for the indicated times. (**d**) Flow cytometric analysis of bacterial load per propidium iodide (PI)-negative iBMDM after infection with GFP-expressing *ΔsifA* Salmonella for the indicated times. Geometric mean of GFP fluorescence (far right panel) from infected cells was normalized to fluorescence at 2 h. (**e**) Fold geometric mean (relative to *Casp11*^*−/−*^ mock complemented control) of fluorescence (representing bacterial load) (10 h/2 h) from indicated iBMDMs after infection with GFP-expressing *ΔsifA* Salmonella. (**f**) Time-lapse microscopy imaging and quantification of GFP-expressing WT or *ΔsifA* Salmonella in C57BL/6 or *Casp11*^*−/−*^ iBMDMs in the presence of PI. When an iBMDM switched from PI-negative to PI-positive, the bacterial load at this time was scored as low (<10 bacteria/cell), med (11–30 bacteria per cell) or high (>30 bacteria per cell) and represented as the % of infected cells (left hand panels). The bacterial load in cells remaining PI-negative at 16 h was then scored (right hand panels) (**g**) Examples of cells quantified in (**f**). White arrows—infected intact iBMDMs; black arrows—infected PI-positive iBMDMs (red). Data represent mean and s.e.m. of three (**a**–**e**) independent repeats or are data from two independent repeats where at least 100 infected cells were scored per experiment (**f**,**g**). Student's *t*-test, *P<0.05, ^**^P<0.01. Scale bar, 20 μm.

To investigate if the apparent intra-macrophage growth defect observed for *ΔsifA* bacteria^27^ is dependent on caspase-11, immortalized bone-marrow derived macrophages (iBMDM) from C57BL/6 and caspase-11 (*Casp11*^*−/−*^) knock-out mice were analysed. There was a greater than 10-fold increase in WT Salmonella over a 12 h time period in C57BL/6 and *Casp11*^*−/−*^ iBMDMs. As expected, far fewer *ΔsifA* bacteria were recovered from C57BL/6 cells; this growth inhibition was partially alleviated in *Casp11*^*−/−*^ (Fig. 3a), but not *Casp7*^*−/−*^ macrophages (Supplementary Fig. 3A). Therefore, as in 3T3 fibroblasts, the low recovery of *ΔsifA* bacteria is at least partially dependent on caspase-11.

To determine if caspase-11 affected macrophage vacuole stability, cytosolic bacteria were quantified by microscopy after selective permeabilisation of the plasma membrane. Less than 5% of WT Salmonella were cytosolic in both C57BL/6 and *Casp11*^*−/−*^ iBMDMs at 2, 6 and 10 h post-uptake (Fig. 3b). At 6 h, ∼25% of *ΔsifA* bacteria were cytosolic in C57BL/6 and *Casp11*^*−/−*^ iBMDMs (Fig. 3b). Therefore, caspase-11 does not affect vacuole escape of *ΔsifA* Salmonella. Whereas the percentage of cytosolic *ΔsifA* bacteria did not increase in C57BL/6 iBMDMs between 6 and 10 h, a significant increase occurred in *Casp11*^*−/−*^ iBMDMs (Fig. 3b). Furthermore, chloroquine-resistant *ΔsifA* bacteria showed a 2-fold growth increase in *Casp11*^*−/−*^ but not C57BL/6 iBMDMs between 7 and 10 h (Supplementary Fig. 3B). Therefore, an increase in the proportion of cytosolic bacteria probably accounts for the increased CFU counts obtained from *Casp11*^*−/−*^ iBMDMs.

In macrophages, caspase-11 initiates cytosolic LPS-dependent cell death^16^,^19^,^23^, characterized by plasma membrane pore formation, cell swelling and lysis, which could result in a reduced number of host cells available for analysis by CFU. In addition, antibiotic present in the culture medium could kill intracellular bacteria after entering host cells through plasma membrane pores^40^. To analyse the extent of caspase-11-dependent cell death following infection with *ΔsifA* Salmonella, *Casp11*^−/−^ iBMDMs were stably transduced with vectors expressing either WT caspase-11 or a catalytically dead mutant^41^ (C_254_G; Supplementary Fig. 3C). As expected, *Casp11*^−/−^ cells expressing WT caspase-11 exhibited greater IL-1β release (Supplementary Fig. 3D) than cells expressing the catalytic mutant. Similarly, caspase-11 catalytic activity was required for cell lysis (indicated by LDH release) up to 6 h post-uptake, when a similar percentage of *ΔsifA* bacteria were cytosolic in the presence or absence of caspase-11 (Fig. 3b,c). However, by 12 h, cell lysis and membrane damage (indicated by uptake of PI) were independent of caspase-11 (Fig. 3c; Supplementary Fig. 3E).

Therefore, the increased CFU counts in macrophages lacking caspase-11 (Fig. 3a) could be attributed (at least in part) to decreased early cell death, but might also involve alleviation of a cytosolic growth inhibitory mechanism. To investigate this further, we measured bacterial loads within individual cells with intact plasma membranes. iBMDMs were infected with GFP-expressing *ΔsifA* bacteria in the presence of PI and were analysed by flow cytometry. By 10 h post-uptake, 8% (±1%) of PI-negative *Casp11*^−/−^ iBMDMs contained a high bacterial load (defined as greater than 750 arbitrary units, corresponding to greater than ∼30 bacteria per cell) compared with 0.4% (±0.1%) in PI-negative C57BL/6 iBMDMs (Fig. 3d, left and centre panels). Analysis of the geometric mean of GFP fluorescence per PI-negative iBMDM revealed a significant increase in *Casp11*^−/−^ iBMDMs at 10 h compared with C57BL/6 iBMDMs (Fig. 3d, right hand panel). Expression in *Casp11*^−/−^ iBMDMs of functional but not of catalytically inactive caspase-11 reduced the intracellular load of *ΔsifA* bacteria at 10 h post-uptake to the level of that observed in C57BL/6 iBMDMs (Fig. 3e). These data suggest that *ΔsifA* bacteria failed to grow in PI-negative WT iBMDMs, at a time when ∼25% of bacteria were cytosolic (Fig. 3b). We then used time-lapse microscopy to provide a more detailed analysis of bacterial growth in intact cells and the onset of PI uptake over time. The bacterial load per cell (represented as the % of infected macrophages containing low, medium or high bacterial loads) was recorded when macrophages switched from PI-negative to PI-positive (Fig. 3f, left hand graph). So that the bacterial load in all cells was recorded, any infected macrophage that remained PI-negative by 16 h post-uptake was also recorded (Fig. 3f, right hand graph). WT *Salmonella* replicated within C57BL/6 iBMDMs over time (Fig. 3f left panel, 3G top panel and Supplementary Movie 1) and occasionally cells became PI-positive (Fig. 3g, black arrow). In agreement with results obtained by flow cytometry (Fig. 3d), *ΔsifA* Salmonella displayed little replication in PI-negative C57BL/6 iBMDMs. Up to 50% of infected cells became PI-positive by 15.5 h, but again the bacterial burden was low (Fig. 3f and Fig. 3g middle panel and Supplementary Movie 2). However, in ∼20% of infected *Casp11*^−/−^ iBMDMs, *ΔsifA* Salmonella had undergone medium (10–30 bacteria/cell) to high (30+ bacteria/cell) levels of replication prior to PI uptake (Fig. 3f right panel, 3G bottom panel and Supplementary Movie 3). This replication might provide an explanation for the increase in LDH release from 6 to 12 h in *Casp11*^−/−^ iBMDMs (Fig. 3c). Furthermore, these time-lapse microscopy experiments reveal (i) a non-synchronous loss of plasma membrane integrity and (ii) caspase-11 mediated growth restriction of *ΔsifA* bacteria in a sub-population of cells that are not PI-positive.

### Caspase-1 and caspase-11 inhibit Δ*sifA* mutant growth

**Figure 4:**
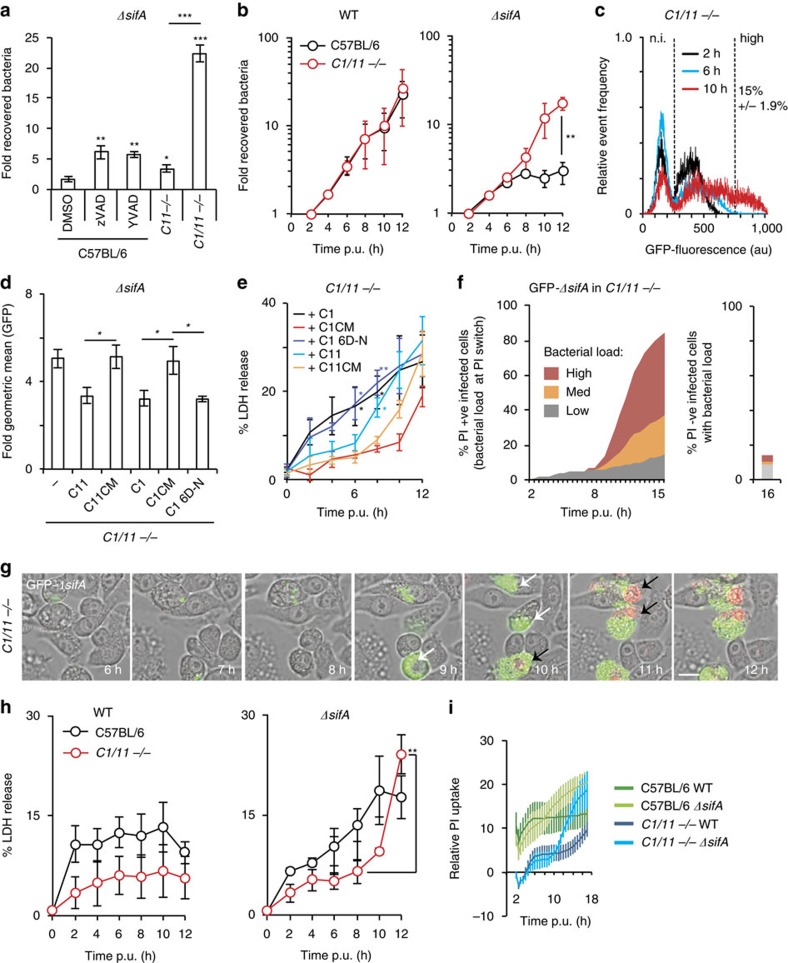
Both caspase-1 and caspase-11 inhibit growth of *ΔsifA* Salmonella. (**a**) C57BL/6 iBMDMs exposed to DMSO, YVAD-FMK or zVAD-FMK, or *Casp11*^*−/−*^ or *Casp1/11*^*−/−*^ iBMDMs, were infected with *ΔsifA* Salmonella for 17 h. CFUs were normalized to bacterial counts at 1 h p.u. (**b**) Kinetics of fold recovered bacterial CFUs for wild-type (WT) and *ΔsifA* Salmonella in C57BL/6 and *Casp1/11*^*−/−*^ (*C1/11−*/−) iBMDMs, normalized to CFUs at 2 h. (**c**) Flow cytometry of PI-negative cells at the indicated times with GFP-expressing *ΔsifA* Salmonella. Data are part of the same experiment as Fig. 3d. (**d**) *Casp1/11*^*−/−*^ iBMDMs expressing caspase-1 (C1), caspase-11 (C11), their catalytic mutants (CM) or a non-cleavable caspase-1 allele (6D-N) (Supplementary Fig. 4B for protein expression) were infected with GFP-expressing *ΔsifA* Salmonella. Bacterial load/cell, expressed as fold geometric mean fluorescence (2 h to 10 h), was determined by flow cytometry of PI-negative cells. (**e**) LDH release from *Casp1/11*^*−/−*^ iBMDMs expressing the indicated proteins and infected with *ΔsifA* Salmonella. Statistical comparisons were made to the samples expressing the corresponding CM allele. (**f**,**g**) Quantification (**f**) and time-lapse imaging (**g**) of *Casp1/11*^*−/−*^ iBMDMs infected with GFP-expressing *ΔsifA* Salmonella in the presence of PI. At the switch time from PI-negative to PI-positive, the bacterial load was scored as low (<10 bacteria /cell), med (11–30 bacteria/cell) or high (>30 bacteria/cell) within that cell, and represented as the % of infected cells (left hand panels). The bacterial load in cells remaining PI-negative at 16 h was then scored (right hand panels). White arrows—intact infected cells, black arrows—PI-positive infected cells (red nuclei). (**h**) Kinetics of macrophage cytotoxicity (LDH release) after infection of indicated iBMDMs with WT or *ΔsifA* Salmonella. (**i**) PI-uptake over time, normalized to 100% cell lysis, in C57BL/6 and *Casp1/11*^*−/−*^ iBMDMs after infection with the indicated Salmonella strains. Data are mean and s.e.m. of at least three independent experiments (**a**–**e**,**h**,**i**) or are data from two independent repeats where at least 100 infected cells were scored per experiment (**f**,**g**). Student's *t*-test (**b**,**e**,**h**) or one-way ANOVA with Dunnett's multiple comparisons test (**a**,**d**) *P<0.05, ^**^P<0.01, ^***^P<0.001. Scale bar, 20 μm.

As alleviation of growth inhibition in *Casp11*^−/−^ iBMDMs was relatively mild, (Fig. 4a) we investigated if other caspase family members might inhibit intracellular bacterial growth. Caspase-1 is constitutively expressed in macrophages but very weakly expressed in the permissive MEF cell type (Fig. 2b, Supplementary Fig. 2A,B), suggesting that caspase-1 might also contribute to growth inhibition of cytosolic bacteria. The involvement of caspase-1 was tested in several ways. First, exposure of iBMDMs to YVAD-FMK (a caspase-1 inhibitor) resulted in increased recovery of *ΔsifA* Salmonella (Fig. 4a) but not WT Salmonella (Supplementary Fig. 4A). Second, significantly more *ΔsifA* bacterial CFUs were recovered from infected *Casp1/11*^−/−^ iBMDMs at 17 h (Fig. 4a) and 12 h post-uptake when compared with *Casp11*^−/−^ iBMDMs (Figs 3a and 4b, for which *Casp11*^−/−^ data were acquired at the same time as *Casp1/11*^−/−^ data). Third, analysis of intracellular *ΔsifA* bacteria by flow cytometry in PI-negative cells revealed that by 10 h post-uptake, 15% of *Casp1/11*^−/−^ iBMDMs contained a high bacterial load (Fig. 4c), compared with 8% of *Casp11*^−/−^ iBMDMs and 0.4% of C57BL/6 iBMDMs (Fig. 3d). As a further test of the contribution of both caspase-1 and caspase-11, WT proteins, catalytically inactive mutants (CM) C1-C_284_A and C11-C_254_G^41^ or a non-cleavable but catalytically active form of caspase-1 (mutated at 6 aspartate residues that become cleaved during autoproteolysis (6D-N)^42^) were expressed individually in *Casp1/11*^−/−^ iBMDMs (Supplementary Fig. 4B). WT but not catalytically inactive caspase-1 and caspase-11 reduced *ΔsifA* bacterial loads significantly (Fig. 4d), implicating both proteases in growth inhibition of cytosolic bacteria. By 6 h, a greater number of *Casp1/11*^−/−^ iBMDMs expressing active caspase-1 underwent *ΔsifA* Salmonella-induced cell death, compared with cells expressing caspase-11. However, by 8 h, caspase-11-dependent cell death was also observed in response to infection by *ΔsifA* (Fig. 4e) but not WT bacteria (Supplementary Fig. 4C). By 12 h, cell death appeared to be independent of caspase-1 and caspase-11 activity (Fig. 4e).

Time-lapse microscopy experiments showed that *ΔsifA* bacteria underwent medium to high levels of replication within *Casp1/11*^−/−^ iBMDMs (Fig. 4f,g and Supplementary Movie 4). This replication, which occurred in ∼80% of infected cells, preceded the uptake of PI and represented a far greater intracellular population than occurred in WT C57BL/6 (<5%) or *Casp11*^*−/−*^ iBMDMs (20%) (Fig. 3f,g). These single-cell analyses provide unequivocal evidence that increased CFU of *ΔsifA* Salmonella in *Casp1/11*^−/−^ iBMDMs was due to enhanced intracellular replication rather than greater survival of infected cells with low bacterial loads.

It was apparent from time-lapse microscopy that the majority of *ΔsifA*-infected *Casp1/11*^−/−^ iBMDMs underwent cell death from 10 h post-uptake (Fig. 4f). Quantification by LDH release (Fig. 4h) and PI uptake (Fig. 4i) confirmed that in *Casp1/11*^−/−^ iBMDMs, cell death was reduced when compared with C57BL/6 iBMDMs for the first 10 h, regardless of the infecting bacterial strain. After this, *Casp1/11*^−/−^ iBMDMs underwent a significantly greater release of LDH and uptake of PI when infected with *ΔsifA* but not WT bacteria (Fig. 4h,i). This is likely to be a non-specific consequence of overwhelming intracellular bacterial growth and/or a cathepsin-dependent cell death that occurs following the enhanced exposure of cytosolic flagellin^43^.

### *ΔsifA* growth arrest does not require cytokine processing

**Figure 5:**
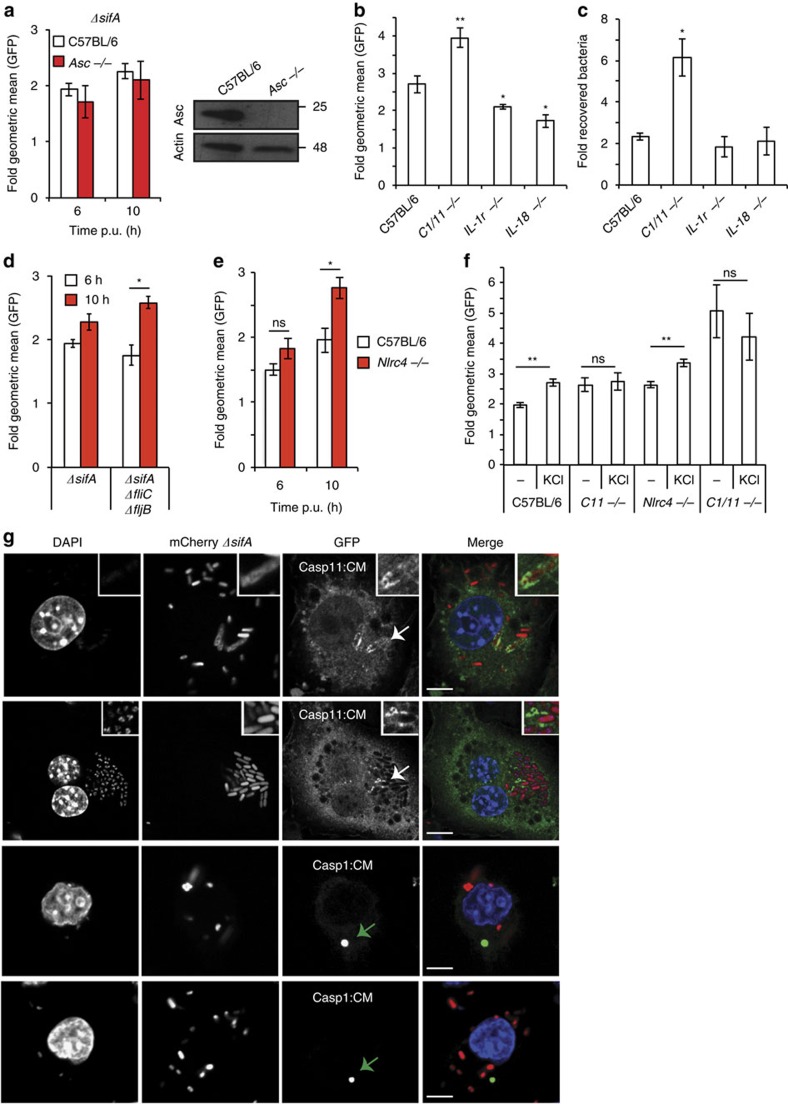
Growth inhibition of *ΔsifA* does not require cytokine production. (**a**,**b**) Flow cytometry analysis of GFP fluorescence per PI-negative cell of the indicated genotype after infection with GFP-*ΔsifA* Salmonella, expressed as fold geometric mean from 2 h. Inset immunoblot for ASC and Actin from the indicated cell lines. (**c**) Fold recovery in *ΔsifA* bacterial CFU at 17 h p.u., normalized to 1 h p.u., from the indicated cell lines. (**d**,**e**) Flow cytometry analysis of GFP fluorescence in the indicated iBMDMs infected with the GFP-expressing bacterial strains expressed as fold geometric mean from 2 h. (**f**) The indicated cell lines were treated with solvent control or KCl and bacterial replication (10 h/2 h) of GFP-*ΔsifA* Salmonella was determined by flow cytometry analysis of GFP fluorescence per PI-negative cell. (**g**) Confocal microscopy images of fixed *Casp1/11*^*−/−*^ iBMDMs expressing GFP-tagged catalytic mutants (CM) of caspase-1 (Casp1:CM) or caspase-11 (Casp11:CM) and infected with mCherry-expressing *ΔsifA* Salmonella for 8 h. DAPI (4′,6-Diamidino-2-Phenylindole, Dihydrochloride) was added to label DNA (blue). White arrows—enlarged boxed area. Green arrows—speck. Mean and s.e.m. of at least three independent repeats (**a**–**f**) or from two representative experiments (**g**). Student's *t*-test, *P<0.05, ^**^P<0.01. Scale bar, 5 μM.

To investigate how caspase-1 and 11 might function we first analysed if pro-inflammatory cytokines were required. Whereas caspase-1-dependent pyroptosis does not require the adaptor protein ASC, efficient secretion of IL-1β and IL-18 requires ASC^44^,^45^ and self-cleavage of caspase-1 (ref. 42). Analysis of *ΔsifA* bacterial loads by flow cytometry showed that growth inhibition did not require ASC (Fig. 5a) or self-cleavage of caspase-1 (Fig. 4d), suggesting that cytokine processing is not required. Indeed, growth attenuation of *ΔsifA* Salmonella was not dependent on IL-18 or IL-1β signalling via the IL-1 receptor (Fig. 5b,c).

Next we analysed whether growth inhibition of *ΔsifA* Salmonella was dependent on the cytosolic receptors NLRC4 and NLRP3. In comparison to *ΔsifA* Salmonella, *ΔsifAΔfljBΔfliC* bacteria underwent small but significant growth between 6 and 10 h within C57BL/6 iBMDMs (Fig. 5d), suggesting that flagella-mediated activation of NLRC4 might contribute to growth inhibitory mechanisms. Indeed, bacterial burden was increased in *Nlrc4*^*−/−*^ iBMDMs (Fig. 5e). The addition of KCl (to inhibit NLRP1/3 activation) further enhanced bacterial growth implicating both NLRP3 and NLRC4 in the growth inhibition of *ΔsifA* Salmonella (Fig. 5f). In contrast, the addition of KCl did not alter the bacterial burden in *Casp11*^−/−^ or *Casp1/11*^−/−^ iBMDMs.

To analyse the cellular localization of caspase-1 and caspase-11 we expressed GFP tagged catalytic mutant proteins in macrophages. As expected, in the majority of *ΔsifA*-infected iBMDMs, GFP-caspase-1CM localized as specks, presumably representing ASC inflammasomes^44^. In contrast, the majority of GFP-caspase-11CM was diffusely cytosolic, but on rare occasions, it was found associated with bacteria (Fig. 5g). The differential localization of caspase-1 and caspase-11 is consistent with independent means of activation and non-redundant roles in growth restriction of cytosolic *Salmonella* (Figs 4 and 5f,g).

### Growth arrest of *ΔsifA* in the absence of Gasdermin D

**Figure 6:**
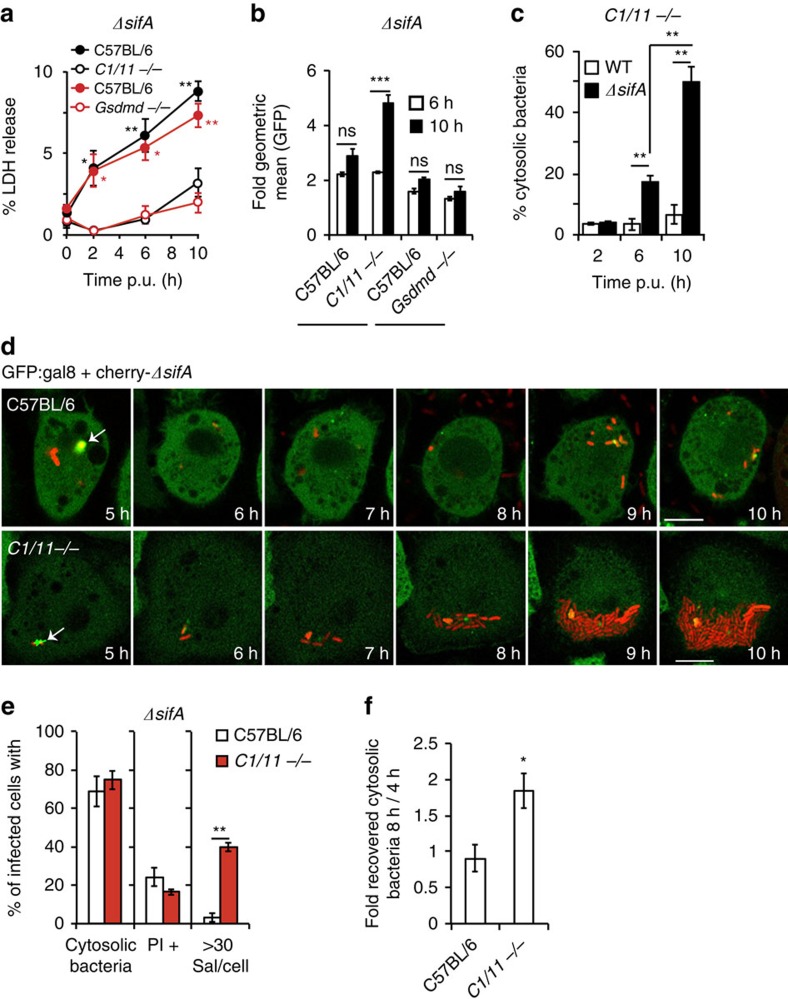
Cytosolic replication of *ΔsifA* in *Casp1/11*^*−/−*^ macrophages. (**a**) Macrophage cytotoxicity was determined by LDH release from the indicated iBMDMs infected with GFP-*sifA* Salmonella. (**b**) Growth of GFP-*sifA* Salmonella in *Gsdmd*^*−/−*^*, Casp1/11*^*−/−*^ and the respective C57BL/6 control iBMDMs. Geometric mean of GFP fluorescence was measured by flow cytometry of PI-negative cells at the indicated time points, normalized to 2 h p.u. (**c**) Quantification of cytosolic WT or *ΔsifA* Salmonella in *Casp1/11*^*−/−*^ iBMDMs by microscopy after selective permeabilisation of the plasma membrane. (**d**) The indicated iBMDMs expressing GFP-tagged galectin-8 and infected with mCherry-expressing *ΔsifA* Salmonella were imaged over time in the presence of PI. White arrows—bacteria associated with ruptured vacuoles. (**e**) *ΔsifA* Salmonella-infected iBMDMs (C57BL/6 or *Casp1/11*^*−/−*^) were quantified for the % of infected cells that harboured cytosolic bacteria, were PI-positive or that contained >30 bacteria per cell at 10 h p.u. (**f**) iBMDMs, infected with WT Salmonella and exposed to chloroquine between 2 and 4 h p.u. were lysed at 4 and 8 h p.u. and bacterial CFUs for cytosolic bacteria determined. Data represent mean and s.e.m. of two (**e**) or three independent experiments (**a**–**c**,**f**). Student's *t*-test, *P<0.05, ^**^P<0.01, ^***^P<0.001. For statistical analysis in (**a**) samples were compared with their respective C57BL/6 control iBMDMs at each time point. Scale bar, 10 μM.

Gasdermin D (Gsdmd) was recently identified as a substrate of caspase-1 and caspase-11 that mediates pyroptotic cell death^46^,^47^. The N-terminal domain of Gsdmd can also kill bacteria *in vitro* directly, but the physiological significance of this activity is unknown^48^. To determine if cleavage of Gsdmd is required for growth inhibition of cytosolic *ΔsifA* Salmonella, *Gsdmd*^*−/−*^ iBMDMs or C57BL/6 control cells from the same source^47^ were infected and bacterial loads determined by flow cytometry. C57BL/6 and *Casp1/11*^*−/−*^ iBMDMs were included in these experiments as controls. Analysis of LDH release confirmed that *Gsdmd*^*−/−*^ iBMDMs, like *Casp1/11*^*−/−*^ iBMDMs, undergo considerably reduced cell death (Fig. 6a). In contrast to *Casp1/11*^*−/−*^ iBMDMs, no significant increase in bacterial burden occurred between 6 and 10 h post-uptake in either C57BL/6 iBMDMs or *Gsdmd*^*−/−*^ iBMDMs (Fig. 6b). Therefore, whereas caspase-1 and caspase-11 activities are required for inhibition of cytosolic growth during this time period, their substrate Gsdmd is not required. Furthermore, the use of *Gsdmd*^*−/−*^ iBMDMs confirms that the absence of cell death alone is not sufficient to enable bacterial replication within the host cell cytosol up to 10 h.

### Replication of *ΔsifA* bacteria in *Casp1/11* ^*−/−*^ iBMDM cytosol

We next investigated if intracellular growth in the absence of caspase-1 and caspase-11 was predominantly cytosolic. Analysis of infected *Casp1/11*^−/−^ iBMDMs following selective permeabilisation of the plasma membrane revealed that, as for C57BL/6 and *Casp11*^*−/−*^ iBMDMs, significantly more *ΔsifA* than WT bacteria were present in the cytosol at 6 h post-uptake (Fig. 6c and Fig. 3b). By 10 h, the percentage of cytosolic *ΔsifA* bacteria had further increased in *Casp1/11*^−/−^ iBMDMs (Fig. 6c), whereas in WT iBMDMs their overall proportion remained relatively unchanged (Fig. 3b). Furthermore, in the presence of chloroquine, *ΔsifA* bacteria underwent replication in *Casp1/11*^−/−^ iBMDMs between 7 and 10 h but not in C57BL/6 iBMDMs (Supplementary Fig. 3B). Time-lapse microscopy of iBMDMs expressing GFP-tagged galectin-8 was used to monitor bacterial replication following vacuole rupture. In agreement with the quantitative analysis (Fig. 3f), C57BL/6 iBMDMs were detected that underwent PI uptake and cell swelling (Supplementary Fig. 5). However, not all WT iBMDMs containing cytosolic bacteria underwent cell death (Fig. 6d, top panel). Within these cells, little replication was observed over the 10 h time period, revealing the presence of an early, cell death-independent inhibition of cytosolic bacterial growth. This was in stark contrast to *Casp1/11*^−/−^ iBMDMs in which dramatic cytosolic replication of *ΔsifA* Salmonella occurred after SCV rupture (Fig. 6d, bottom panel). Altogether with whole population replication assays (Fig. 4b,c), these results reveal that *ΔsifA* Salmonella undergo extensive cytosolic replication following SCV rupture in the absence of both caspase-1 and caspase-11.

The relative contribution of cell-death dependent and independent control of cytosolic bacterial growth was then assessed at the whole population level by analysing the percentage of infected cells containing cytosolic bacteria, as well as plasma membrane integrity and bacterial load. Approximately 70% of *ΔsifA*-infected C57BL/6 iBMDMs contained at least 1 cytosolic bacterium by 10 h post-uptake (Fig. 6e). At this time, 25% of iBMDMs had become PI-positive. Therefore, even if all PI-positive cells contained cytosolic bacteria, of the remaining PI-negative cells, at least half must have contained cytosolic bacteria that had not undergone significant growth. In contrast, 40% of *Casp1/11*^−/−^ iBMDMs contained a high bacterial load (>30 bacteria/cell), despite a similar number of *Casp1/11*^−/−^ cells harbouring cytosolic bacteria as C57BL/6 iBMDMs (Fig. 6e). These results show that inhibition of cytosolic bacterial growth can occur prior to cell death and that this requires the activities of caspase-1 and caspase-11.

### Caspase-1 and -11 repress growth of cytosolic WT Salmonella

In the course of these experiments we detected a small population (<5%) of cytosolic WT Salmonella (Figs 3b and 6c). A chloroquine protection assay was used to kill vacuolar bacteria, enabling analysis of this subpopulation in the presence or absence of caspase-1 and caspase-11. In *Casp1/11*^−/−^ iBMDMs exposed to chloroquine, twice as much growth of cytosolic WT bacteria occurred between 4 and 8 h compared with C57BL/6 cells (Fig. 6f). Altogether with our findings in 3T3 fibroblasts (Fig. 1), these results indicate that both caspase-1 and caspase-11 contribute to cytosolic growth inhibition of WT Salmonella.

### Effects of caspases on *ΔsifA* bacteria in primary macrophages

**Figure 7:**
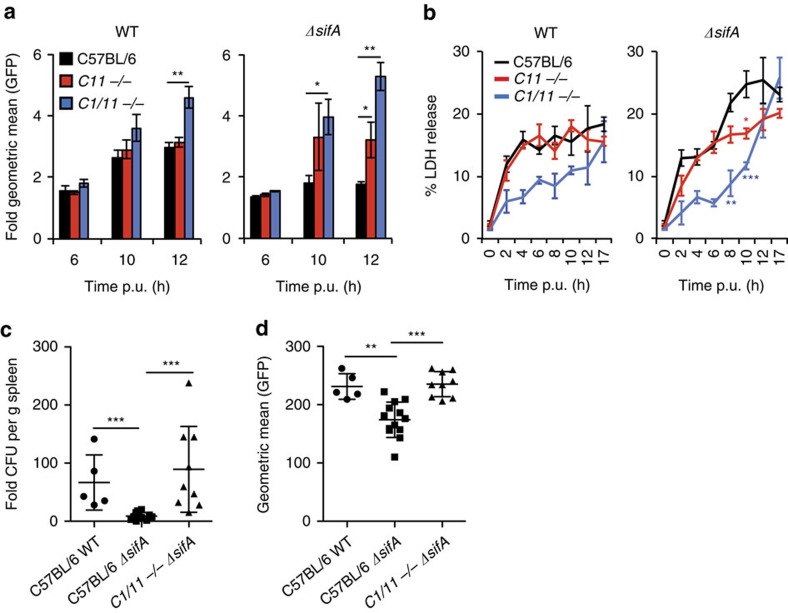
Caspase-mediated inhibition of *ΔsifA* in primary macrophages. (**a**) Growth of GFP-WT or GFP-*sifA* Salmonella in primary BMDMs isolated from C57BL/6 mice, caspase-11 knockout mice (*C11*^*−*/−^) or caspase-1 and caspase-11 double knockout mice (*C1/11*^*−*/−^). Geometric mean of GFP fluorescence was measured by flow cytometry of PI-negative cells at the indicated time points, normalized to 2 h p.u. (**b**) Macrophage cytotoxicity was determined by LDH release from the indicated primary BMDMs infected with GFP-WT or GFP-*sifA* Salmonella. (**c**,**d**) C57BL/6 or *Casp1/11*^*−*/−^ mice were infected with GFP-WT or GFP-*sifA* Salmonella for 48 h. Bacterial CFUs/g spleen (**c**) were calculated from the inoculum and the geometric mean of GFP fluorescence per CD11b(+) splenic cells was measured by flow cytometry (**d**). Each symbol represents one mouse with the mean and standard deviation represented. Data represent mean and s.e.m. of three independent experiments (**a**,**b**). Student's *t*-test, *P<0.05, ^**^P<0.01, ^***^P<0.001. For statistical analysis in (**b**) samples were compared with LDH release from C57BL/6 BMDMs.

To determine if primary bone-marrow-derived macrophages (BMDM) inhibit growth of cytosolic Salmonella, bacterial loads were measured by flow cytometry in PI-negative cells. Similar to our observations in iBMDMs, primary BMDMs inhibited growth of *ΔsifA* Salmonella in a caspase-1 and caspase-11-dependent manner (Fig. 7a). Furthermore, by 12 h, the burden of WT Salmonella had increased in the *Casp1/11*^−/−^ BMDMs. At 8 h and 10 h post-uptake, LDH release following infection by *ΔsifA* Salmonella was dependent on both caspase-1 and caspase-11. However, from 12 h onwards, cell death was independent of caspase-1 and 11 (Fig. 7b), similar to our findings in immortalized BMDMs (Figs 3c and 4h,i). Therefore, our results with immortalized cells are reflected in primary cells and unlikely to be an artefact of the immortalization process. Finally, we examined bacterial loads in splenocytes obtained from C57BL/6 and *Casp1/11*^−/−^ mice at 48 h following intraperitoneal inoculation of GFP-expressing Salmonella strains. As expected, in C57BL/6 mice *ΔsifA* Salmonella were severely attenuated for overall growth compared with WT bacteria and this defect was rescued in *Casp1/11*^−/−^ mice (Fig. 7c). Analysis of bacterial loads by flow cytometry revealed far fewer *ΔsifA* Salmonella in CD11b(+) macrophages compared with WT Salmonella (Fig. 7d). However, macrophages from *Casp1/11*^−/−^ mice harboured numbers of *ΔsifA* Salmonella that were similar to those of WT bacteria in CD11b(+) cells from C57BL/6 mice (Fig. 7d), indicating caspase-1 and 11-dependent growth inhibition of cytosolic *Salmonella in vivo*.

## Discussion

In the present work we analysed the fate of host cells and cytosolic bacterial growth at both whole population and single cell levels. Our two major findings are that (1) cells undergo a heterogeneous response upon bacterial infection: over a time course of several hours, not all cells containing cytosolic bacteria undergo cell lysis, and even in cells that lyse, the timing of loss of plasma membrane integrity varies widely. (2) Intracellular cytosolic bacterial growth can be inhibited either before or independently of the onset of host cell death; this process requires activity of both caspase-1 and 11. Control of cytosolic bacterial growth also involves the receptors NLRC4 and possibly NLRP3. In contrast, the absence of Gsdmd was not sufficient to alleviate growth attenuation up to 10 h. Furthermore, caspase-mediated processing of cytokines did not appear to be required as the absence of the adaptor protein ASC, the cytokine IL-18 or the receptor for IL-1β (IL-1r) did not yield increased growth of cytosolic bacteria.

The Salmonella *sifA* mutant provides a convenient if artificial means to expose bacterial surface ligands to cytosolic receptors and to analyse the fate of cytosolic bacteria in macrophages. However, following phagocytosis, a small proportion of WT bacteria also lose their vacuolar membranes (Fig. 3b)^9^, and we found that caspase-1 and caspase-11 inhibited their cytosolic growth in both immortalized and primary macrophages. Therefore, the experiments involving *ΔsifA* Salmonella are applicable to WT bacteria, which could be potentially very detrimental to the host if they were to replicate in the nutrient-rich macrophage cytosol. The importance of caspase-mediated growth attenuation of cytosolic Salmonella was also revealed in non-phagocytic cells, where SPI-1 T3SS-dependent invasion results in a greater proportion of cytosolic bacteria. In addition, lack of growth inhibition in MEFs and inhibition of growth in 3T3 fibroblasts were directly correlated with the absence and presence of caspase-11, respectively. Increased cytosolic replication of Salmonella in human colonic epithelial cells following knock-down of caspase-4 was reported by Knodler *et al*.^24^,^34^. This was attributed to delayed shedding of host cells; however loss of a cytosolic growth inhibition might also have contributed to this phenotype.

Previous studies have shown that caspases prevent cytosolic growth of Salmonella^23^,^24^ and *Legionella*^49^ through pyroptosis. In the latter case, degradation of cytosolic bacteria was also observed, and this was reduced in cells exposed to zVAD-FMK. However, it is not clear if cells containing degraded bacteria were intact or undergoing cell death. In agreement with several previous experiments^13^,^16^,^18^,^23^ we found that not all cells that were exposed to cytosolic Salmonella undergo cell death. Several experiments showed that by 10 h post-uptake of *ΔsifA* Salmonella in C57BL/6 macrophages, cell death ranged from 20 to 30%, even though ∼70% of cells contained cytosolic bacteria (Figs 3c,f and 6e). If the macrophage cytosol is normally permissive for bacterial growth, then growth would be expected to occur in the cytosol of non-pyroptotic cells. However, by analysing bacterial load in single cells by flow cytometry and time-lapse microscopy in the presence of PI, it was clear that bacterial growth could be inhibited prior to the loss of plasma membrane integrity. In contrast, in the absence of caspase-1 and caspase-11 the macrophage cytosol was very permissive for bacterial growth, indicating additional functions for these inflammatory caspases. In support of this, Gsdmd, which is required for pyroptosis^46^,^47^, did not contribute significantly to inhibition of bacterial growth up to 10 h. This provides compelling evidence that the macrophage cytosol can restrict bacterial growth and that caspase-1 and caspase-11 have functions beyond the onset of cell death to mediate this activity.

Other lines of evidence support a dual role for caspases in the control of cytosolic Salmonella. First, although WT and *ΔsifA* bacteria induced similar levels of cell death in 3T3 fibroblasts, growth of *ΔsifA* Salmonella only occurred after caspase inhibition (Fig. 1a,b). Second, expression of caspase-11 in MEFs was insufficient to elicit cell death (presumably due to the absence of other infection-induced proteins required for pyroptosis^9^,^26^,^50^) but nevertheless reduced bacterial numbers significantly (Fig. 2). Interestingly, caspase activation without concomitant cell death has also been reported to occur in Salmonella-infected neutrophils^51^. Non-pyroptotic caspase-mediated bacterial growth inhibition has previously been reported for vacuolar bacteria: caspase-1 regulates macrophage phagosome acidification (thereby contributing to killing of *Staphylococcus aureus*^31^) and it promotes *Legionella* phagosome fusion with lysosomes^30^,^52^. Caspase-11 also appears to have additional functions, enhancing lysosomal fusion of *Legionella* vacuoles through modulation of cofilin, a regulator of actin polymerization^29^,^53^. Finally, *Casp1/11*^*−/−*^ iBMDMs produce reduced mROS and hydrogen peroxide, required for effective control of vacuolar Salmonella^32^. However, these mechanisms are unlikely to explain our findings on cytosolic Salmonella as caspase inhibition would be expected to influence vacuolar WT bacteria to a similar or greater extent.

Autophagy can inhibit bacterial growth following the rupture of pathogen-containing vacuoles in epithelial cells^7^,^36^. In addition, members of the guanylate-binding protein family control intracellular bacterial growth by pyroptotic and non-pyroptotic mechanisms, including antibacterial autophagy and the induction of bacterial cell lysis by an unknown mechanism^9^,^54^,^55^. Autophagy is unlikely to account for the activities we described here for the following reasons: *ΔsifA* Salmonella are not targeted to the autophagic machinery in HeLa cells^7^ and we detected a similar level of association of the autophagy marker LC3B to Salmonella after the addition of zVAD-FMK (Supplementary Fig. 1C).

Therefore, our results suggest that additional substrate(s) of both caspase-1 and caspase-11 generate the production of antimicrobial activity within the cytosol before or without the onset of pyroptosis, adding to the increasing roles of these caspases beyond pyroptosis and cytokine processing. Several studies have identified putative caspase-1 substrates^56^,^57^ including transcription factors, cytoskeletal components and glycolytic enzymes. Whether cytosolic antimicrobial activity might be due to an antimicrobial peptide, such as ubiquicidin^58^, limited cellular glycolysis when caspase-1 is active or through modulation of the cytoskeleton as described for vacuolar bacteria^29^,^32^,^52^ awaits further investigation. The growth of *sifA* mutant Salmonella remained attenuated in *Gsdmd*^*−/−*^ iBMDMs up to 10 h, but it is possible that direct GSDMD-mediated killing of bacteria^48^ could occur at later time points. Mechanistically, it is noteworthy that self-cleavage of caspase-1, which is not required for cell death^42^ is also not required for restriction of bacterial growth, highlighting the different functions of cleaved and uncleaved caspase-1. This suggests that substrates of processed caspase-1, such as pro-IL-1β and IL-18 are insufficient to explain our results. In line with this, the absence of ASC did not result in enhanced growth of the *sifA* mutant and IL-1r^−/−^ or IL-18^−/−^ iBMDMs were still able to control replication of *ΔsifA* Salmonella.

Many factors could account for the heterogeneous response to cytosolic bacteria. These include concentrations and/or availability of appropriate ligand, sensor, caspase enzyme and its substrate(s) all of which could vary from cell to cell. In addition, variation in caspase-11 levels could result from differential transcriptional upregulation after priming by agonists including LPS.

Whereas caspase-11 can be activated through direct binding to cytosolic LPS (ref. 18), infection with Salmonella also activates caspase-1 via the NLRP3 and NLRC4 inflammasomes^44^. In this respect, absence of NLRC4 partially alleviated growth inhibition of the *sifA* mutant, which was further alleviated by exposure to KCl. This could represent a requirement for NLRP1 or NLRP3 but as C57BL/6 macrophages have been shown to have dysfunctional NLRP1b (ref. 59), it seems more likely that NLRP3 is involved. In *Casp11*^*−/−*^ macrophages, the addition of KCl did not significantly alter intracellular bacterial growth, suggesting non-canonical NLRP3 activation. Although caspase-11 contributes to the release of IL-1α, its cell death-inducing function appears to be independent to that of caspase-1 (refs 13, 20, 60). Our evidence indicates that caspase-1 and caspase-11 also function independently in their cell autonomous bacterial growth-suppressive activities. Growth of cytosolic Salmonella was significantly greater in *Casp1/11*^*−/−*^ compared with *Casp11*^*−/−*^ macrophages. In addition, microscopic analysis of infected cells also suggested independent activities of caspase-1 and caspase-11: caspase-1 was found predominantly in single inflammasome ‘specks' following infection whereas caspase-11 was diffusely cytosolic and infrequently (<5%) associated with bacteria. Altogether, this suggests that caspase-1 and caspase-11 could employ distinct mechanisms to restrict bacterial replication within the cytosol.

The relative contributions of caspase-1 and caspase-11 in the control of cytosolic Salmonella have been analysed following mixed infections of WT and *sifA* mutant bacteria in WT, *Casp11*^*−/−*^ and *Casp1/11*^*−/−*^ knock-out mice^23^. The equivalent competitive index values of the *sifA* mutant in *Casp11*^*−/−*^ and *Casp1/11*^*−/−*^ backgrounds suggested a major role for caspase-11 but not caspase-1 in the growth inhibition of *sifA* mutant bacteria. However, loss of caspase-1-mediated growth attenuation of WT Salmonella^16^ could have masked an effect of caspase-1 on *sifA* mutant bacteria within the mixed infection. If so, this would be consistent with our results that show a clear function for both caspase-1 and caspase-11 in the growth control of cytosolic *ΔsifA* Salmonella.

In conclusion, our experiments have revealed a surprising degree of heterogeneity in the response of host cells to cytosolic bacteria. We found that the catalytic activities of both caspase-1 and caspase-11 function to control growth of cytosolic Salmonella by both pyroptotic and non-pyroptotic mechanisms. Since vacuoles containing pathogenic or commensal bacteria can be ruptured through pathogen or host-dependent mechanisms^9^, caspase-dependent cytosolic growth inhibitory activity could prevent a wide variety of bacteria from cytosolic replication.

## Methods

### Antibodies and reagents

Antibodies for immunoblotting were from Sigma (actin, AC-74 used at 1:5,000 dilution and caspase-11, 17D9 used at 1:1,000 dilution), Cell Signaling (caspase-7, 9492 used at 1:1,000 dilution), Adipogen (caspase-1 p20, AG-20B-0042 used at 1:1,000 dilution), DSHB (tubulin, E7 used at 1:5,000 dilution) and Santa Cruz Biotechnology (ASC sc-22514-R used at 1:1,000 dilution). Propidium iodide was from Life Technologies and Lipofectamine2000 from Invitrogen. siRNAs for caspase-7 and caspase-11 were purchased from Santa Cruz and used at 40 pmol. zVAD-FMK and YVAD-FMK were from R&D systems.

### Bacterial infections

*Salmonella enterica* serovar Typhimurium (strain 12023) was grown overnight in LB. GFP-expressing Salmonella carry plasmid pFPV25.1, mCherry-expressing Salmonella carry plasmid pDiGc (ref. 61). *prgH* mutant Salmonella carry the plasmid pRI203, expressing *Yersinia* InvA (ref. 62). Bacteria (20 μl) were opsonized with 20 μl mouse serum (Sigma) in 170 μl DMEM for 20 min before addition of 600 μl DMEM. Macrophages (in 500 μl media in 24 well plates) were infected with 40 μl of opsonized bacteria (MOI 5–10), centrifuged at 110 *g* for 5 min and incubated for 25 min at 37 °C. Following two washes with PBS, cells were incubated with 100 μg ml^−1^ gentamicin for 2 h and then 20 μg ml^−1^, or directly incubated with 20 μg ml^−1^ gentamicin. For SPI-1 T3SS-mediated invasion of 3T3 fibroblasts or MEFs, stationary phase bacterial cultures were sub-cultured (1:33) in fresh LB and grown for 3.5 h at 37 °C before inoculation. Cells in 24 well plates (500 μl media/well) were infected with 7 μl of sub-cultured bacteria for 7 min. After two PBS washes cells were incubated with 100 μg ml^−1^ gentamicin for 2 h and 20 μg ml^−1^ gentamicin thereafter.

### Cell culture

C57BL/6 WT, *Asc*^*−/−*^, *Casp11*^*−/−*^, *Casp1/11*^*−/−*^, *Casp7*^*−/−*^, IL-18^−/−^, IL-1r^−/−^ and *Nlrc4*^*−/−*^ BMDM were infected with the v-myc/v-raf expressing J2 retrovirus^63^, and differentiated in 20% L929-MCSF supernatant. Cells were then maintained in Dulbecco's modified Eagle medium (DMEM, Sigma), 10% fetal calf serum (FCS), 20% L929-MCSF and 1 mM sodium pyruvate at 37 °C, 5% CO_2_. 3T3 fibroblasts, MEFs, 293ETs and RAW 264.7 macrophages (ATCC) were cultured in DMEM containing 10% FCS. C57BL/6 control and *Gsdmd*^*−/−*^ iBMDMs were maintained as described above. Cell lines, tested for mycoplasma, were chosen for ease of Salmonella infection, enabling analysis after both invasive and non-invasive uptake. Primary BMDMs were differentiated in 20% L929-MCSF supernatant for 1 week after isolation. For assays investigating the effect of caspase inhibitors, cells were incubated in DMEM with 10% FCS supplemented with 20 μM zVAD-FMK, 20 μM YVAD-FMK or 20 μM of other peptide inhibitors (Supplementary Fig. 1D) or DMSO (1:1000) as vehicle control, for 1 h before infection. When indicated KCl was added at 50 mM, 1 h before infection.

### Constructs and retroviral transductions

Plasmids encoding GFP-tagged galectin-8 and LC3B were kind gifts from Dr Felix Randow and have previously been described^36^. Genes encoding murine caspase-1 or caspase-11 were ligated into a replication-defective retroviral plasmid (m6p) (ref. 64). Site directed mutagenesis was used to introduce mutations, which were verified by sequencing. Caspase-1 6D-N comprises 6 Asp to Asn mutations, preventing self-cleavage, while maintaining catalytic activity^42^. For transduction, retroviral particles were packaged into vesicular stomatitis virus pseudotyped virus after co-transfection of 293ET cells. After 48 h, cells were selected in puromycin (2.5 μg ml^−1^) or blasticidin (5 μg ml^−1^) so that all cells within a population expressed the transgene. Where GFP fusions were used, cells were sorted by Fluorescence-Activated Cell Sorting to obtain a 100% GFP-positive population.

### Colony forming unit assay

To enumerate intracellular bacteria, cells from duplicate or triplicate wells of a 24 well plate, infected as above, were lysed in 1 ml of ice cold PBS containing 0.1% Triton X100 for 5 min. Serial dilutions were plated on duplicate LB agar and plates were incubated overnight at 37 °C. Colonies were counted using an Acolyte colony counter. Where CQ treatment was used (Sigma, 250 μM) it was added between 1.5 and 3 h (3T3 fibroblasts) or 2 and 4 h (WT infected iBMDMs) or 6 and 7 h (*ΔsifA* Salmonella). For 3T3 fibroblasts the colony counts are represented as the fold growth in vacuolar bacteria (total—CQ resistant) and cytosolic bacteria (CQ resistant). For iBMDMs the fold growth in CQ-resistant bacteria (cytosolic) are shown.

### ELISA

Concentrations of IL-1β in macrophage culture supernatants were measured using mouse IL-1β kits according to manufacturer's recommendations (Affymetrix ebioscience) after uptake of Salmonella

### Flow cytometry

To measure the replication of GFP-expressing Salmonella in intact cells, cells were infected as above and harvested following trypsin treatment, washed and re-suspended in Optimem (Invitrogen) containing 1 μg ml^−1^ Propidium Iodide (PI). Data, consisting of at least 10,000 events, were acquired on a FACs Calibur and analysed using FlowJo 8.8.6. Data are represented as the fold-change (from 1 or 2 h p.u.) in geometric mean of cells harbouring GFP-expressing bacteria.

### Immunoblotting

Proteins in post nuclear supernatants from 1 × 10^6^ cells were separated on either 10% or 12% Tris polyacrylamide gels. Proteins were transferred to Nitrocellulose membranes, which were then blocked in 5% milk in TBST (100 mM Tris Cl pH 7.4, 150 mM NaCl, 0.1% Tween20). Membranes were incubated overnight at 4 °C with primary antibodies, washed three times with TBST and then incubated for 2 h with secondary antibodies at room temperature. Visualization was done using ECL+detection regents (GE Healthcare). Uncropped blots are shown in Supplementary Fig. 6.

### LDH cytotoxicity assay

Host cell death was measured as a percentage of total LDH release, according to the recommended protocol (Promega). Medium was used as a blank control to obtain background measurements and supernatants from non-infected samples were subtracted from infected conditions. Total LDH release was measured after cell lysis at −80 °C.

### Mice

For primary BMDMs, Caspase-1/11 double knockout mice were from the Swiss Immunological Mouse Repository (SwImMR) and caspase-7^−/−^ mice were purchased from Jackson Laboratories. *Casp11*^*−/−*^ primary BMDMs had been isolated from previously described mice^65^. C57BL/6 control mice were from Charles Rivers. All animals were bred in accordance with accredited animal facility regulations at Imperial College London. Imperial College Animal Welfare and Ethical Review Body (AWERB) granted approval for all mouse work. iBMDMs, that have been previously described were prepared from *Nlrc4*^*−/−*^*, Asc*^*−/−*^ (ref. 45), *Casp1/11*^*−/−*^ (ref. 66) and *Casp11*^*−/−*^ (ref. 65) mice. For mouse infections, mice (8–10 week old, C57BL/6 or *Casp1/11*^*−/−*^) were inoculated intraperitoneal with 1 × 10^5^ CFUs with GFP expressing WT or *ΔsifA* Salmonella. After 48 h, spleens were collected, homogenized and splenic CD11b(+) cells enriched using magnetic beads according to the manufacturer instructions (Miltenyi Biotec). Purified cells were analysed by flow cytometry in Optimem containing PI. Mice showing very poor infection of the spleen were excluded. Randomization and blinding were not used.

### Microscopy and digitonin assays

Cells were seeded on glass cover slips one-day prior to infection and fixed in 4% paraformaldehyde for 20 min. Confocal images were taken on a Zeiss 710 microscope with a × 100 objective. For digitonin-mediated permeabilisation of the plasma membrane, live cells were treated with 40 μg ml^−1^ digitonin for 5 min on ice prior to immunolabelling with anti-CSA1 (1:400, Kirkegaard and Perry Laboratories), anti-GM130 (1:500, BD Transduction laboratories) and anti-PDI (Protein disulfide-isomerase, 1:100, Enzo) for 30 min on ice. Cells were then washed twice in PBS and fixed in 4% paraformaldehyde. After permeabilisation in PBS, 0.1% Triton X100 and 10% horse serum, cover slips were incubated with appropriate AlexaFluor secondary antibodies (Invitrogen) and DAPI (4′,6-Diamidino-2-Phenylindole, Dihydrochloride) for 30 min before mounting onto glass slides.

### PI uptake

PI uptake was used to determine plasma membrane integrity. Macrophages (3 × 10^5^ cells per ml) were seeded in white clear-bottomed 96-well plates (Greiner) and infected with opsonised late stationary phase Salmonella (MOI 10:1) for 30 min at 37 °C. Following infection, cells were washed twice with PBS and 200 μl Optimem medium containing 10% FCS, 20 μg ml^−1^ gentamicin and 1 μg ml^−1^ PI was added. Triton X-100 (0.1%) was included in Optimem medium in wells used for positive controls. Optimem medium without PI was added to negative control wells. Plates were incubated at 37 °C in 5% CO_2_ within a Tecan Infinite M200PRO fluorescent plate reader throughout infection, with PI fluorescence measured every 15 min. Non-infected controls were subtracted from infected samples and then divided by the fluorescence of wells treated with Triton-X100 to give the relative PI uptake.

### Quantitative reverse transcriptase (RT)-PCR

Total RNA was isolated from 1 × 10^6^ cells (Qiagen RNAeasy mini kit) and 400 ng was used to synthesize complementary DNA (cDNA) according to manufactures recommendations (QuantiTect RT kit, Qiagen). cDNA (0.5 μl) was used in quantitative RT-PCRs (SybrGreen PCR master mix, Applied biosystems) containing 0.2 μM gene-specific primers. After determining the cycle threshold (Ct) required to reach a significant emission of Sybr Green reporter dye (Rotor-Gene 3000, Corbett Research), relative mRNA was calculated from a titration curve of cDNA. Data represent the relative amounts of mRNA, normalized to rps9 house keeping gene. The following primers were used:

Caspase-1 For 5′- ACTGGGACCCTCAAGTTTTG,

Rev 5′- CATCTCCAGAGCTGTGAG

Caspase-7 For 5′-TGGAAAAGGTGGATTCTTCC,

Rev 5′-CTTTGTCGAAGTTCTTGTTG

Caspase-11 For 5′-AAACACCCTGACAAACCACT,

Rev 5′-TTCCTCCATTTCCAGATTAG

Rps9 For 5′- CTGGACGAGGGCAAGATGAAGC-3′

Rev 5′- TGACGTTGGCGGATGAGCACA-3′

### Time-lapse microscopy

Cells seeded in dishes (Matek) with an embedded glass cover slip were infected as above. Prior to imaging, medium was replaced with Optimem (Invitrogen) containing 10% FCS, 40 μM Hepes (Sigma), 20 μg ml^−1^ gentamicin and 1 μg ml^−1^ PI. Cells were maintained at 37 °C in a heated chamber and images were acquired at 20-min intervals using the × 40 objective on a Zeiss 710 confocal microscope. At least 100 infected cells were scored per experiment.

### Statistics

Either an one-way ANOVA with Dunnett's multiple comparisons test or one and two-tailed unpaired equal variance Students *t*-test were used for statistical comparison from three or more independent experiments as indicated. *P<0.05, **P<0.01, ***P<0.001. Experiments were performed with at least three independent replicates, except for the analysis of time-lapse microscopy. When possible, pilot data with a type I error rate of 5% was used to determine an appropriate sample size.

### Data availability

The authors declare that the data supporting the findings of this study are available within the article and its Supplementary Information Files.

## Additional information

**How to cite this article:** Thurston, T. L. M. *et al*. Growth inhibition of cytosolic Salmonella by caspase-1 and caspase-11 precedes host cell death. *Nat. Commun.* **7,** 13292 doi: 10.1038/ncomms13292 (2016).

**Publisher's note:** Springer Nature remains neutral with regard to jurisdictional claims in published maps and institutional affiliations.

## Supplementary Material

Supplementary Information Supplementary Figures 1-6

Supplementary Movie 1 Time lapse imaging of C57BL/6 iBMDMs from 2 hours post-uptake with GFP-WT Salmonella in the presence of Propidium iodide (red). Images were acquired every 15 minutes on a Zeiss 710 confocal microscope with the 40x objective.

Supplementary Movie 2 Time lapse imaging of C57BL/6 iBMDMs from 2 hours post-uptake with GFP-ΔsifA Salmonella in the presence of Propidium iodide (red). Images were acquired every 15 minutes on a Zeiss 710 confocal microscope with the 40x objective.

Supplementary Movie 3 Time lapse imaging of Casp11-/- iBMDMs from 2 hours post-uptake with GFP-ΔsifA Salmonella in the presence of Propidium iodide (red). Images were acquired every 15 minutes on a Zeiss 710 confocal microscope with the 40x objective.

Supplementary Movie 4 Time lapse imaging of Casp1/11-/- iBMDMs from 2 hours post-uptake with GFP-ΔsifA Salmonella in the presence of Propidium iodide (red). Images were acquired every 15 minutes on a Zeiss 710 confocal microscope with the 40x objective.
