# Supplementary material for: Growth inhibition of cytosolic Salmonella by caspase-1 and caspase-11 precedes host cell death
Source: Nat Commun. 2016 Nov 3;7:13292. doi: 10.1038/ncomms13292 (PMC5097160; doi:10.1038/ncomms13292)
Supplement: Supplementary Information — Supplementary Figures 1-6 [file ncomms13292-s1.pdf]

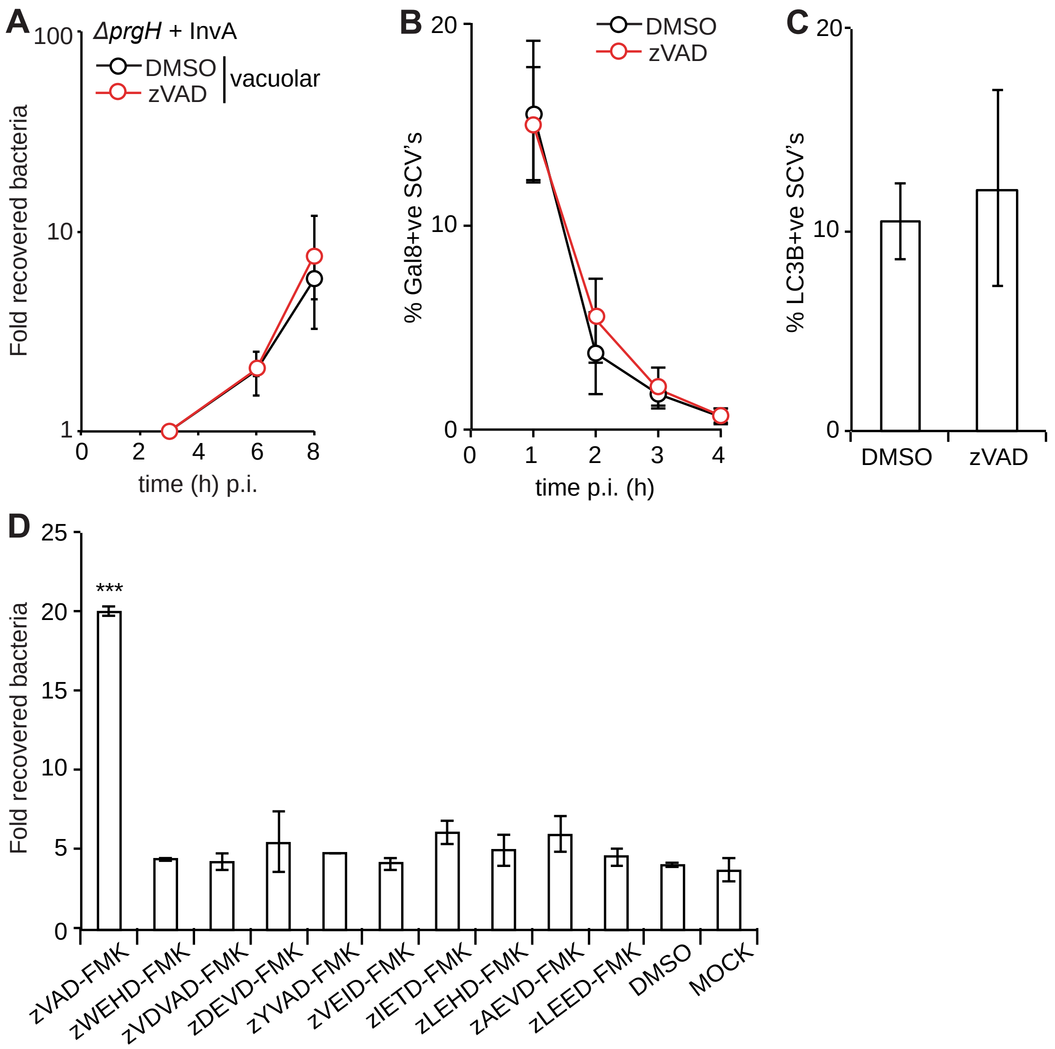

Supplementary Figure 1: Inhibition of caspase activity does not alter SCV rupture

(A) DMSO or zVAD-FMK treated 3T3 fibroblasts were infected with *ΔprgH* Salmonella expressing *Yersinia* invasion (InvA) and treated with solvent control or chloroquine from 1.5 – 3 h post invasion (p.i.). The numbers of surviving vacuolar bacteria (total population – CQ resistant population) was determined by CFU. (B) The percentage of Salmonella within ruptured vacuoles was scored in 3T3 fibroblasts expressing GFP-galectin-8 after infection with mCherry-expressing WT Salmonella in the presence of DMSO or zVAD-FMK. (C) 3T3 fibroblasts expressing GFP-LC3B were treated as in (B) and analysed at 1 h p.i. (D) Fold bacterial replication (8 h / 2 h) in 3T3 fibroblasts treated with the indicated peptide inhibitors was determined by plating CFUs after cell lysis.

Data are the mean and SEM from three experiments (A-D). One-way ANOVA with Dunnett’s multiple comparisons test, *** $P<0.001$.

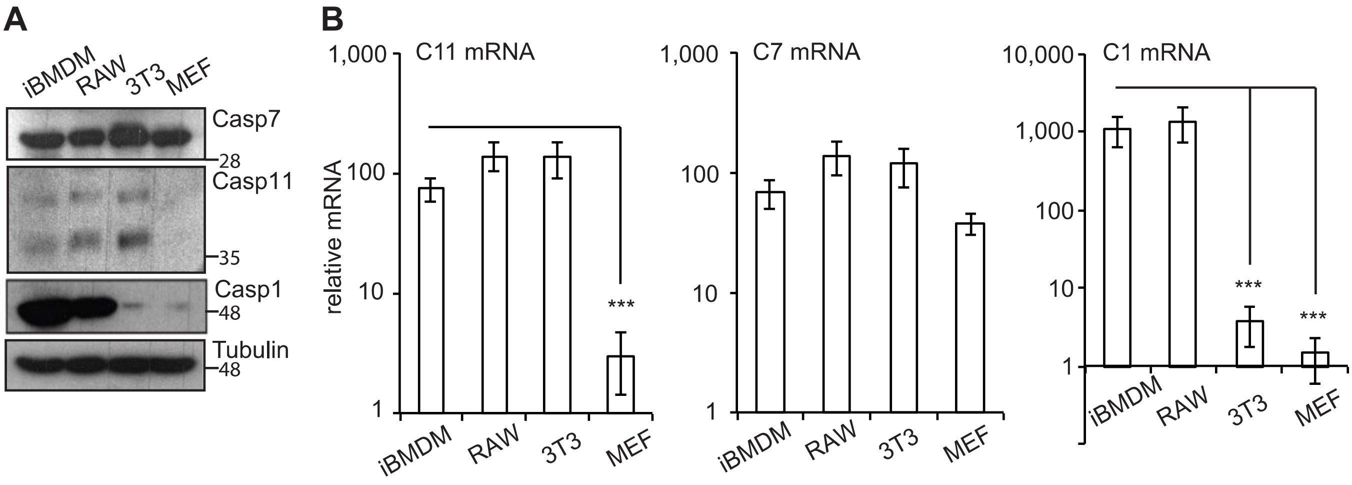

Supplementary Figure 2: Cell type variation in caspase expression

(A) Protein extracts from the indicated cell lines immunoblotted for caspase-7 (casp7), caspase-11 (casp11), caspase-1 (casp1) or Tubulin as a control. (B) Analysis of mRNA levels for caspase-11 (C11), caspase-7 (C7) or caspase-1 (C1) by quantitative RT-PCR in uninfected cells.

Data are representative of three experiments (A) or are the mean and SEM of three independent repeats (B). Student's *t*-test, ****P*<0.001

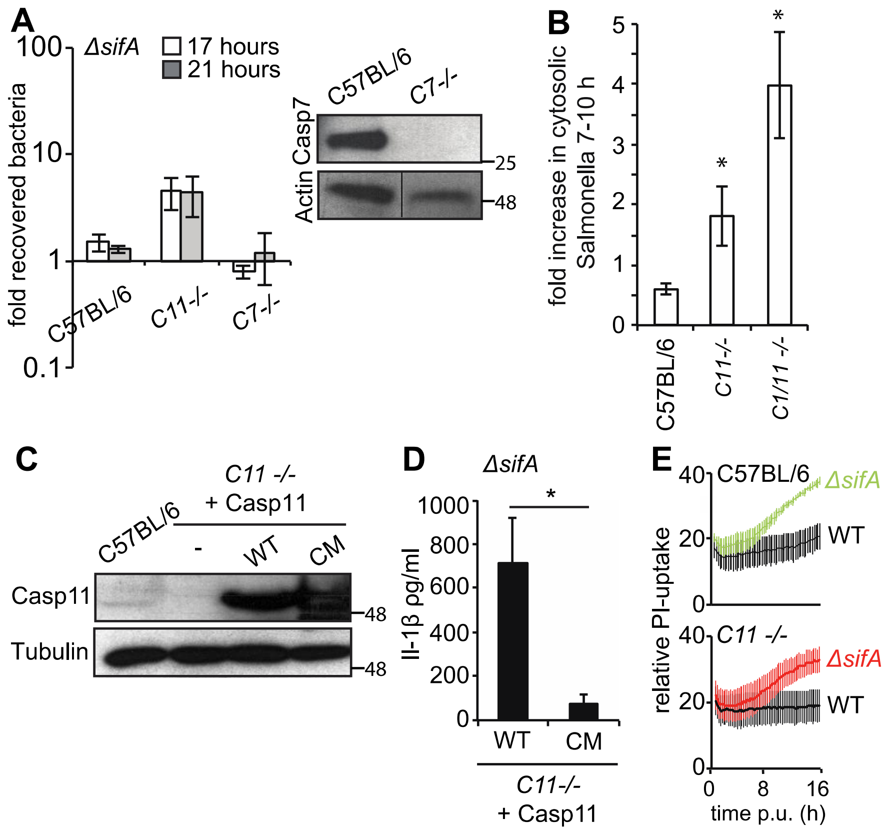

Supplementary Figure 3: Caspase-11 but not caspase-7 inhibits *ΔsifA* growth

(A) Enumeration of *ΔsifA* Salmonella CFU on LB medium following lysis of infected C57BL/6, *Casp11*$^{-/-}$ (*C11-/-*) and *Casp7*$^{-/-}$ (*C7-/-*) iBMDMs at 17 and 21 hours p.u.. Inset immunoblot probed with anti-caspase-7 and anti-Actin antibodies. (B) Indicated iBMDMs were treated with chloroquine (CQ) from 6 – 7 h post uptake. The numbers of surviving *ΔsifA* cytosolic bacteria (CQ-resistant population) were determined by CFU at 7 and 10 hours and represented as fold increase from 7 h. (C) Caspase-11 immunoblot of *Casp11*$^{-/-}$ iBMDMs expressing wild type (WT) or catalytically inactive (CM) caspase-11 after retroviral transduction, together with mock transduced C57BL/6 and *Casp11*$^{-/-}$ iBMDMs. (D) Amounts of IL-1β released from *Casp11*$^{-/-}$ iBMDMs expressing wild-type or CM caspase-11 and infected with *ΔsifA* Salmonella (10 h). (E) PI-uptake in C57BL/6 and *Casp11*$^{-/-}$ iBMDMs analysed over time by an automated fluorescence plate reader after

infection with the indicated Salmonella strains. Data were normalized to a control for 100% PI-positive cells.

Data are mean and SEM of three experiments (A, B, D and E) or representative of two independent experiments (C). Student's *t*-test, *P<0.05.

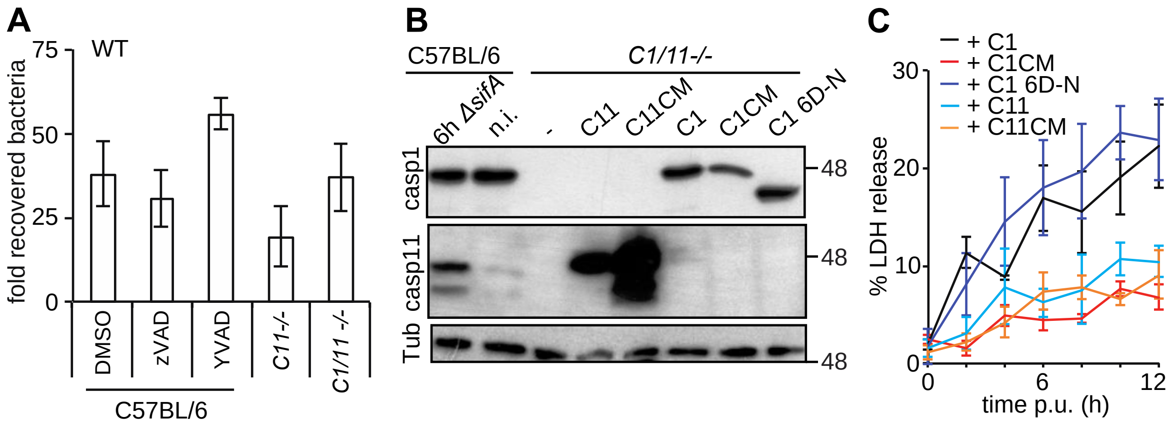

Supplementary Figure 4: Analysis of WT Salmonella within macrophages

(A) Fold recovery in WT bacterial CFU at 17 h p.u., normalized to 1 h p.u., from C57BL/6 iBMDMs treated with DMSO, zVAD-FMK or YVAD-FMK, or *Casp11*$^{-/-}$ or *Casp1/11*$^{-/-}$ iBMDMs (B) Levels of caspase-11 (C11), caspase-1 (C1), catalytic mutants (C11CM, C1CM) or a non-cleavable caspase-1 (C1 6D-A) in *Casp1/11*$^{-/-}$ iBMDMs after retroviral transduction. Cells lysates were immunoblotted for caspase-1 (casp1), caspase-11 (casp11) or Tubulin (Tub) from non-infected cells, except where indicated. (C) Kinetic analysis of LDH release in *Casp1/11*$^{-/-}$ iBMDMs expressing the indicated proteins and infected with WT Salmonella. Mean and SEM of three experiments (A and C) or from two representative experiments (B).

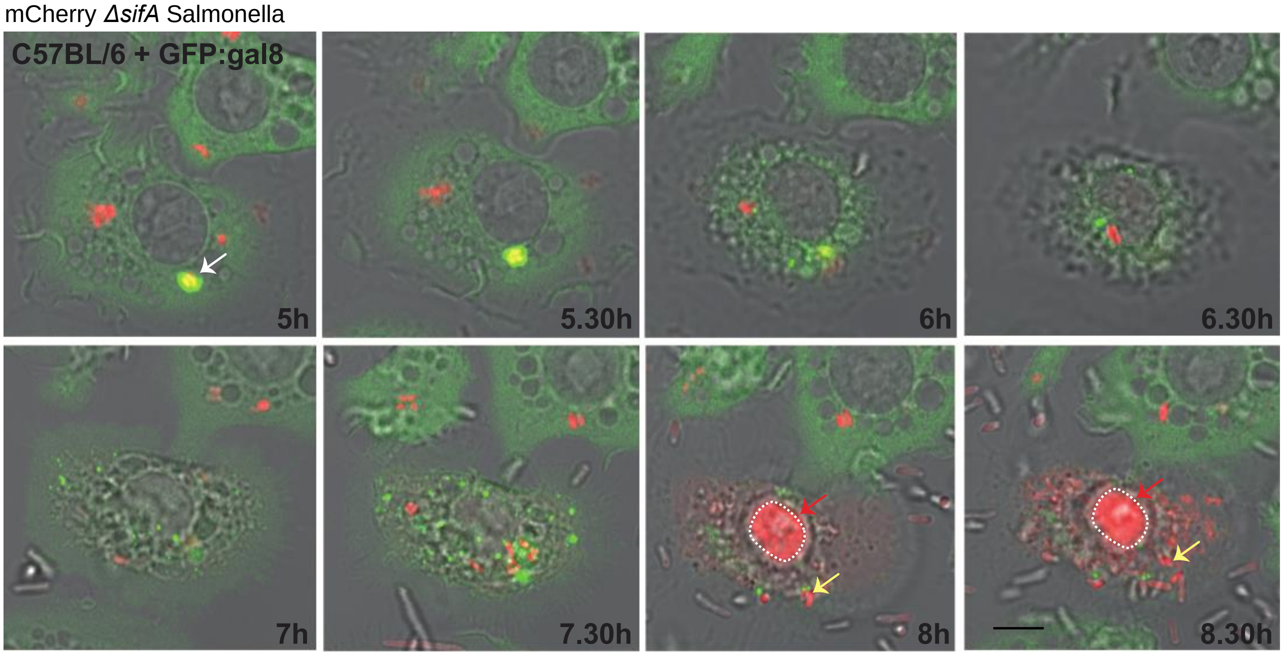

Supplementary Figure 5: Time-lapse microscopy in GFP-galectin-8 macrophages

(A) C57BL/6 iBMDMs expressing GFP-tagged galectin-8 and infected with mCherry-expressing *ΔsifA* Salmonella in the presence of propidium iodide (red) were imaged at the indicated times. White arrow – bacteria associated with ruptured vacuole. Red arrows - PI-positive nuclei (red surrounded by white dotted line). Yellow arrows – example mCherry-expressing *ΔsifA* Salmonella.

Scale bar 5 μM.

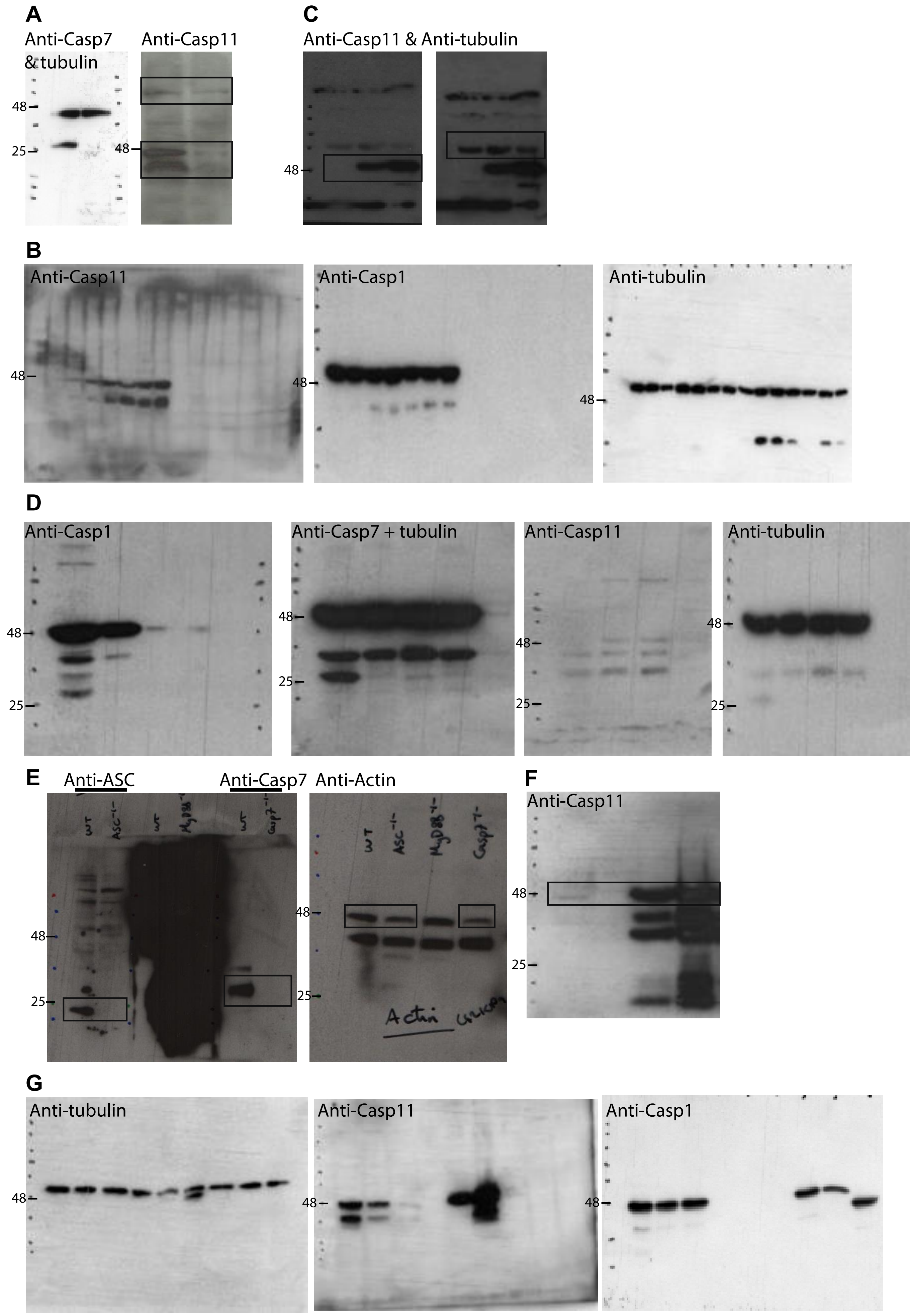
A
Anti-Casp7 & tubulin
Anti-Casp11
48
25
48
C
Anti-Casp11 & Anti-tubulin
48
B
Anti-Casp11
Anti-Casp1
Anti-tubulin
48
48
48
D
Anti-Casp1
Anti-Casp7 + tubulin
Anti-Casp11
Anti-tubulin
48
25
48
25
48
25
48
25
E
Anti-ASC
Anti-Casp7
Anti-Actin
48
25
48
25
WT
ASC-/-
MyD88-/-
Casp7-/-
Actin
F
Anti-Casp11
48
25
G
Anti-tubulin
Anti-Casp11
Anti-Casp1
48
48
48

Supplementary Figure 6: Full western blots

Uncropped blots corresponding to those shown in Figures 1G (A), 2B (B) and 2D (C), Supplementary Fig. 2A (D) and 3A and Fig. 5A (E), Supplementary Fig. 3C (F) and 4B (G) of the manuscript. When present, boxes are the cropped bands shown for the figures.
